# Breath and Beyond: Advances in Nanomedicine for Oral and Intranasal Aerosol Drug Delivery

**DOI:** 10.3390/ph17121742

**Published:** 2024-12-23

**Authors:** Simeng Du, Zhiyang Wen, Jinghan Yu, Yingying Meng, Yuling Liu, Xuejun Xia

**Affiliations:** 1 State Key Laboratory of Bioactive Substance and Function of Natural Medicines, Institute of Materia Medica, Chinese Academy of Medical Sciences & Peking Union Medical College, Beijing 100050, China; dusimeng@imm.ac.cn (S.D.); wenzhiyang@imm.ac.cn (Z.W.); yujinghan@imm.ac.cn (J.Y.); mengyingying@imm.ac.cn (Y.M.); ylliu@imm.ac.cn (Y.L.); 2 Beijing Key Laboratory of Drug Delivery Technology and Novel Formulation, Institute of Materia Medica, Chinese Academy of Medical Sciences & Peking Union Medical College, Beijing 100050, China

**Keywords:** OIADD, nano drug delivery system (NDDS), formulation and preparation process, aerosolization device

## Abstract

Designing and standardizing drug formulations are crucial for ensuring the safety and efficacy of medications. Nanomedicine utilizes nano drug delivery systems and advanced nanodevices to address numerous critical medical challenges. Currently, oral and intranasal aerosol drug delivery (OIADD) is the primary method for treating respiratory diseases worldwide. With advancements in disease understanding and the development of aerosolized nano drug delivery systems, the application of OIADD has exceeded its traditional boundaries, demonstrating significant potential in the treatment of non-respiratory conditions as well. This study provides a comprehensive overview of the applications of oral and intranasal aerosol formulations in disease treatment. It examines the key challenges limiting the development of nanomedicines in drug delivery systems, formulation processes, and aerosol devices and explores the latest advancements in these areas. This review aims to offer valuable insights to researchers involved in the development of aerosol delivery platforms.

## 1. Introduction

Oral and nasal aerosolized drug delivery (OIADD) is a method for delivering medications directly to the respiratory system in aerosolized form through the oral and nasal cavities. In the pharmaceutical field, the U.S. Pharmacopeia and National Formulary (USP-NF) classifies oral and nasal aerosolized drug delivery under the same category, “Orally inhaled and Nasal drug products”, which emphasizes the commonality of quality and product performance testing methods. This categorization emphasizes the commonality of quality and product performance testing methods. Moreover, based on the high degree of overlap in formulary design, manufacturing, and control of these two kinds of products, the U.S. Food and Drug Administration’s (FDA) guidance on drug manufacturing, “Nasal Spray and Inhalation Solution, Suspension, and Spray Drug Products--Chemistry, Manufacturing, and Controls”, was adopted by the U.S. Food and Drug Administration (FDA). Products-Chemistry, Manufacturing, and Controls Documentation” also discusses the two together.

The pharmaceutical industry is currently undergoing rapid advancement, which has led to the emergence of OIADD as a key area of interest. This is due to the unique ability of OIADD to deliver drugs directly to targeted sites in the lungs, nasal cavity, or brain while also facilitating rapid absorption into the bloodstream via the highly vascularized nasal mucosa or alveoli, resulting in rapid therapeutic effects. OIADD presents significant advantages over traditional oral and injectable delivery methods. The oral route of administration is frequently hindered by gastrointestinal instability and first-pass effects, thereby significantly reducing drug bioavailability. Despite being a direct method, injectable delivery has several disadvantages, including the potential for pain, infection risks, and the inconvenience of prolonged treatment regimens. In contrast, OIADD has the advantage of minimizing systemic side effects and enhancing drug concentrations at the target site, which makes it particularly effective under respiratory and nasal conditions. Furthermore, as a non-invasive treatment, OIADD enhances patient compliance and provides an optimal solution for repeated administration and self-management with significant clinical applications. In the realm of respiratory disease, aerosolized medications have emerged as the preferred treatment for a number of conditions. Furthermore, OIADD demonstrates substantial potential in the treatment paradigm of neurological disorders, including Alzheimer’s and Parkinson’s diseases, as well as other conditions such as dry eye and diabetes. The growing focus on cardiovascular disease treatment further highlights the extensive applicability and substantial research and development potential of OIADD beyond respiratory diseases.

Incorporating nanomedicine into the research context of OIADD further underscores its transformative potential. Despite the significant advantages of OIADD, its development faces numerous challenges, including poor drug solubility, limited physiological barrier penetration, and short drug retention times. Effective therapeutic strategies for nasal and pulmonary delivery remain scarce, as drugs often struggle to penetrate deep into the lower respiratory tract. Multidrug resistance, chronic inflammation, mucus barriers, and bacterial biofilm formation further compromise therapeutic efficacy. Nanomedicine integrates nano drug delivery systems (NDDS), advanced nanodevices, and innovative formulation processes into a unified framework to enhance therapeutic efficacy, precision, and patient outcomes, overcoming the limitations of traditional therapies. Notably, NDDS leverages its nanoscale size, high surface area, and versatile surface functionalization to address the key limitations of conventional treatments while meeting the demands of future disease therapies. However, aerosolizing NDDS for inhalation introduces unique challenges, such as ensuring aerosol stability, effective deposition, and redispersibility—issues that are distinct from other drug delivery methods. Advancing OIADD requires not only innovations in NDDS design but also improvements in formulation processes and aerosolization devices. Optimizing these aspects is critical for enhancing drug delivery efficiency, improving patient convenience, and fully realizing the potential of OIADD in both research and clinical applications.

This review is rooted in the forefront of nanomedicine, focusing on the application of OIADD in disease treatment, covering respiratory, brain, cardiovascular, and other multisystem diseases. It provides a detailed account of the strategies used by NDDS to overcome the limitations of OIADD, along with the latest research advancements. Furthermore, it offers an in-depth analysis of the impact of formulation processes and aerosolization devices on NDDS. The review highlights new achievements and technologies in OIADD, including advanced NDDS designs, formulation optimization, and the development of novel aerosolization devices, providing guidance for future research in this field.

## 2. OIADD in the Treatment of Diseases

### 2.1. Disorders of the Respiratory System

The history of aerosol therapy can be traced back to ancient times, with the earliest known reference dating back to the ancient Egyptian Ebers Papyrus, approximately 1554 BCE. This document describes the use of roasted scopolamine leaves to produce smoke that can be inhaled for the treatment of respiratory difficulties. The Yellow Emperor’s Classic of Internal Medicine documents the use of ephedra by the Chinese to treat asthma. Ancient Assyrian cuneiform texts describe the inhalation of chromones for asthma treatment. Additionally, ancient Indians inhaled mandrake and marijuana to treat respiratory disorders [1]. With advancements in science and technology, the development of OIADDs for the treatment of respiratory diseases has become increasingly sophisticated, leading to the availability of a wide range of products in the market.

#### 2.1.1. Nasal Disorders

Intranasal glucocorticoid aerosol preparations are the first line of therapy for allergic rhinitis. Owing to their poor dosage precision and drug stability [2], they are typically used only for mildly localized conditions or for nasal cavity cleansing. Although multidose nasal drops are still available in the market, they are considered outdated because they require preservatives, which can cause some degree of damage to the nasal mucosa. In addition, improper head positioning can result in swallowing a portion of the medication being swallowed [3]. To improve nasal deposition, users may need to adopt specific positions, such as tilting the head back and extending the neck, to allow droplets to deposit under the influence of gravity [4,5]. Aerosol-generating nasal sprays are gradually replacing nasal drops because of their ability to deliver a stable dose and their ease of use. A wide variety of nasal sprays are available on the market, including nasal corticosteroids (e.g., beclomethasone propionate: Beconase^®^, GlaxoSmithKline and Qnasl^®^, Teva, flunisolide: Nasalide^®^, Teva, budesonide: Rhinocort^®^, Johnson & Johnson Consumer, fluticasone: Flonase^®^, Haleon and Xhance^®^, Optinose, mometasone: Nasonex^®^, Perrigo Pharma, ciclesonide: Omnaris^®^, Covis, and the azelastine–fluticasone combination: Dymista^®^, Mylan), nasal antihistamines (e.g., azelastine: Astepro^®^, Mylan, olopatadine: Patanase^®^, Novartis), and nasal anticholinergics (e.g., ipratropium bromide: Atrovent^®^, Boehringer Ingelheim). Aerosol-generating nasal sprays are currently the most commonly used dosage forms for treating nasal cavity disorders [6].

#### 2.1.2. Lung Disorders

Bronchial asthma and chronic obstructive pulmonary disease (COPD) are obstructive airway diseases. The Global Initiative for Asthma (GINA) and Global Initiative for Chronic Obstructive Lung Disease (GOLD) guidelines identify oral inhalation formulations as the first-line therapy for both bronchial asthma and COPD [7,8]. Traditionally, β_2_-agonists are combined with glucocorticoids for inhalation therapy in asthma, such as Airsupra^®^ (Budesonide/Salbutamol, AstraZeneca) for asthma prevention and rescue, and Symbicort^®^ (Budesonide/Formoterol Fumarate, AstraZeneca) and Dulera^®^ (Formoterol Fumarate/Mometasone Fumarate, Organon) for maintenance treatment. For COPD, after dual inhalation therapy (β_2_-agonist/Muscarinic receptor antagonist), GOLD guidelines recommend triple therapy (β2-agonist/Muscarinic receptor antagonist/glucocorticoid) if symptoms acutely worsen. Breztri Aerosphere^®^ (Budesonide/Formoterol Fumarate/Glycopyrronium Bromide, AstraZeneca), approved in 2020, is based on positive results from phase III clinical trials. Compared to Bevespi Aerosphere^®^ (Formoterol Fumarate/Glycopyrronium Bromide, AstraZeneca), Breztri Aerosphere^®^ significantly reduced the rate of moderate or severe acute exacerbations in COPD patients by 24% [9]. In addition, the emergence of dual-action drugs offers new options for the treatment of COPD. For example, Ohtuvayre^®^ (Ensifentrine, Verona Pharma) is an oral inhaled phosphodiesterase 3/4 (PDE3/4) inhibitor, which exerts a synergistic effect by simultaneously inhibiting PDE3 and PDE4 enzymes, thereby reducing inflammation and improving bronchodilation with a single compound [10]. In the future, as more clinical trials and new drugs are developed, the use of inhaled agents in obstructive airway disease will become more widespread and precise, leading to more efficient treatment options.

In the early phases of inhalation therapy for pulmonary infectious diseases, clinicians often aerosolize antibiotics that were originally intended for intravenous injection with the objective of increasing the concentration of the drug at the site of infection. However, this approach has the potential to irritate the respiratory tract and cause subsequent airway inflammation [11]. Numerous inhaled antibiotic formulations have been developed with progress in aerosolization technology. For example, Tobramycin solution Tobi^®^ (Tobramycin, Novartis) (TIS) was the first antibiotic approved for inhalation therapy in patients with cystic fibrosis (CF). Although TIS showed excellent tolerability and improved lung function, it required lengthy nebulization times and storage at 2~8 °C. The later introduction of the inhaled dry powder formulation, Tobi^®^ Podhaler^®^ (Tobramycin, Novartis) (TIP), offered similar therapeutic efficacy with a 112 mg dose, one-third of the 300 mg dose required for TIS [12]. Additionally, the administration time of TIP is at least one-third that of TIS, and TIP can be stored at room temperature. Another notable advancement is the inhaled lyophilized powder Cayston^®^ (Aztreonam, Gilead), which inhibits the growth of over 90% of Pseudomonas aeruginosa isolates in CF patients with minimal systemic absorption. This has effectively replaced aerosolized aztreonam injections in clinical practice [13].

According to the literature, traditional intravenous pulmonary arterial hypertension treatments increase heart rate and cardiac output while reducing left atrial and systemic blood pressure [14]. In contrast, aerosolized products such as the inhalation solution Ventavis^®^ (Iloprost, Schering), Tyvaso^®^ (Treprostinil, United Therapeutics), or the inhaled dry powder Yutrepia^®^ (Treprostinil, Liquidia) have a smaller impact on systemic hemodynamics, thereby reducing systemic side effects. These treatments also improve gas exchange by dilating the arteries that supply well-ventilated areas of the lungs.

Orally inhaled medications are currently used as adjunctive therapies in clinical practice. For example, the etiology of acute respiratory distress syndrome (ARDS) is complex, and its treatment strategy has remained primarily supportive, with no single therapy showing a clear clinical benefit [15,16]. The limited effectiveness of systemic treatments for ARDS can be attributed to several factors [17,18,19]. First, the diverse causes of ARDS make it difficult to demonstrate the efficacy of a “standardized” treatment regimen; second, patients with ARDS suffer from multiple organ failure and are unable to tolerate the off-target effects of drugs. In an analysis of tissue fibrinolytic drugs delivered by different modes of administration, researchers found that aerosolized administration resulted in greater fibrinolytic activity in plasma and bronchoalveolar lavage fluid (BALF) compared to intravenous administration. Moreover, aerosolized fibrinolytics effectively reduced alveolar neutrophils, whereas intravenous or intraperitoneal administration did not significantly reduce alveolar neutrophils [20]. In addition, there is a lack of effective clinical treatment options to completely cure idiopathic pulmonary fibrosis (IPF). The Japanese Respiratory Society proposed the most recent international treatment guidelines in 2023, which generally recommend a combination of oral pirfenidone or nintedanib with inhaled N-acetylcysteine for IPF treatment [21]. In other cases, in the treatment of tuberculosis (TB), current oral treatment regimens typically last 6~9 months [22]; however, the therapeutic dose reaching the lesion site is often inadequate, leading to the regrowth of “persistent” bacteria and increased drug resistance. Moreover, lung cancer, one of the most common malignant tumors worldwide, is treated with surgery, radiotherapy, chemotherapy, and immunotherapy [23]. However, systemic administration of drugs, currently the main treatment for both diseases, not only has significant side effects but also suffers from problems of patient compliance and drug resistance, especially in the course of long-term therapy [24]. Therefore, in the future, oral-inhaled drug delivery may bring a turnaround to the inadequacy of systemic drug delivery for the treatment of these diseases because of the advantage of direct action on lesions.

### 2.2. Brain Disorders

Various treatment methods are available for brain disorders, including invasive delivery approaches such as intracerebral, intraventricular, and intrathecal injections. Although these methods provide direct access, they are invasive and pose significant health risks. Oral administration is the most common treatment modality. However, adverse effects of orally administered drugs and in-depth studies of the “gut-brain axis” have shown that brain disorders tend to “implicate” the gastrointestinal system and leave it vulnerable [25]. For instance, patients with migraines frequently experience nausea, vomiting, and gastrointestinal discomfort, which significantly reduce their compliance with oral medications [26]. Similarly, patients with Parkinson’s disease (PD) suffer from delayed gastric emptying and gastroparesis, complicating the absorption of oral drugs, with additional complications arising from Helicobacter pylori infections and the impact of food [27]. In comparison, pulmonary drug delivery takes advantage of the large surface area of the alveoli to rapidly transport drugs into the bloodstream and subsequently across the blood–brain barrier (BBB) to reach the brain [28]. More strikingly, nose-to-brain delivery does not require crossing the BBB and entering the central nervous system (CNS) through three main pathways: the olfactory mucosa at the top of the olfactory region, the trigeminal pathway, and the mucosal epithelial pathway of the olfactory region, which allows for the delivery of therapeutic drugs directly to the brain in a highly targeted manner [29,30,31,32].

For PD treatment, Inbrija^®^, the first FDA-approved orally inhaled formulation of levodopa, has demonstrated faster symptom relief than oral administration. A clinical study showed that inhaled levodopa reaches its T_max_ in approximately 15 min, which is significantly shorter than the 45 min to 2 h required for traditional oral administration, depending on food intake [33].

Triptans are first-line drugs for the treatment of migraines in moderate-to-severe cases [34]. Initially, triptans were introduced in tablet and injectable forms; subsequently, they were developed as nasal sprays for the rapid relief of acute migraine. The first triptan nasal spray, Imitrex^®^ (Sumatriptan, GlaxoSmithKline), was launched in 1997, with a nasal bioavailability of 17% compared to intramuscular injection. To improve bioavailability, Tosymra^®^ (Sumatriptan, Upsher-Smith Laboratories), a formulation containing sumatriptan and a permeation enhancer, was approved in 2019. Tosymra^®^ achieved an absolute bioavailability of 87.7% via nasal administration [35]. Compared to Imitrex^®^, Tosymra^®^ is absorbed more rapidly, with a C_max_ of 63.9 ng/mL for 10 mg Tosymra^®^ and 21.4 ng/mL for 20 mg Imitrex^®^, and an AUC_0–2h_ of 48.4 ngh/mL versus 24.7 ngh/mL, respectively [36]. Triptans exert their effects by antagonizing 5-hydroxytryptamine receptors expressed in the cerebral and coronary arteries, which can pose risks to patients with cardiovascular diseases [34]. In contrast to triptans, calcitonin gene-related peptide (CGRP) is a neuropeptide that plays a pivotal role in the trigeminal system and is used in the treatment of migraines. Early CGRP receptor antagonists are available as oral formulations and have common side effects, including nausea and vomiting. In 2023, Pfizer introduced Zavzpret^®^, the first CGRP receptor antagonist approved for use as a nasal spray. In a phase III, double-blind, randomized, placebo-controlled multicenter trial, Zavzpret^®^ exhibited its efficacy in the acute treatment of migraines, accompanied by favorable tolerability and safety. It begins to take effect within 15 min, with pain relief lasting up to 48 h [37], making it a new option for patients with cardiovascular diseases or those unable to take oral medications [38].

In the field of epilepsy treatment, the FDA approved the nasal spray Nayzilam^®^ (Midazolam, UCB) for marketing in 2019. As an alternative to traditional injectable administration, Nayzilam^®^ has a T_max_ of 9.0~21.5 min, peak sedation effect within 15~120 min after administration, and an intranasal bioavailability of approximately 80% of that of intravenous administration, providing a new option for patients with cluster seizure epilepsy over the age of 12 years. Due to its rapid onset of action and ease of administration, Nayzilam^®^ is particularly suitable for on-the-spot resuscitation of acute seizures [39]. In January 2020, diazepam nasal spray was approved by the FDA to be marketed in the United States under the trade name Valtoco^®^ (Diazepam, Neurelis). Although it has been shown that the bioavailability of 20 mg intranasal diazepam is comparable to that of the marketed 20 mg diazepam rectal gel [40], intranasal administration may be readily preferred by patients due to better compliance.

### 2.3. Other Disorders

With continuous advancements in medical research, OIADD has begun to show unique value and promise for other treatments. Table 1 summarizes the key OIADD products currently approved by the FDA for the treatment of various diseases (excluding those withdrawn from the market). These examples demonstrate that OIADD provides patients with noninvasive and convenient treatment options for different diseases. As more OIADD products are developed and marketed, this field is anticipated to advance treatment strategies for various diseases, offering patients additional therapeutic options and increasing convenience.

In addition, OIADD has shown significant potential for the treatment of cardiovascular diseases. Earlier, researchers found that Ultrafine particulate matter in polluted air can lead to endothelial damage, systemic inflammation, arrhythmias, and cardiac dysfunction [41,42]. Given the pulmonary circulation, researchers have begun exploring the possibility of delivering medications to the heart through the inhalation of aerosolized drugs, potentially replacing invasive parenteral routes, such as intravenous, intracoronary, pericardial, or intramyocardial injections, currently used to treat heart diseases [43]. Miragol et al. [44] compared the advantages of respiratory aerosolized nanoparticles over the three routes of oral (gavage), intraperitoneal, and intravenous administration for targeting the heart. They prepared calcium phosphate (CaP) nanoparticles with a diameter of approximately 50 nm, labeled them with the fluorescent dye cyanine 7 (Cy7), and administered them to healthy mice. Fluorescence detection and in vivo optical imaging revealed that aerosolized CaP-Cy7 nanoparticles showed the highest accumulation in the heart. Additionally, they found that inhaled CaP-Cy7 demonstrated better cardiac targeting than Cy7 alone without crossing the blood–brain barrier, eliciting immune responses, or negatively affecting cardiac function. Therefore, inhaled CaP-Cy7 may offer improved efficacy and safety for the treatment of heart-related diseases. Liu et al. [45] utilized Cy5-labeled synthetic ROS-scavenging nanoparticles (Cy5/TPCD NPs) to investigate their distribution in lung cells and blood lymphocytes in a mouse model of heart failure to elucidate the primary mechanism of nanoparticle transfer from the lungs to the heart. Flow cytometry results revealed that 25.6 ± 1.9% of epithelial cells and 29.7 ± 5.6% of lung endothelial cells were Cy5-positive, with significant Cy5 expression observed in lung epithelial and endothelial cells, while less than 5% of lymphocytes exhibited Cy5 positivity. This suggests that lymphatic transport is not the predominant pathway for cardiac accumulation of Cy5/TPCD NPs. Instead, these nanoparticles are likely transported from the pulmonary capillaries to the heart via the lung epithelium and endothelium, which is theoretically the shortest route between the lungs and heart [46]. These findings indicate that transoral aerosols represent a promising new approach for targeted drug delivery to the heart and offer a novel avenue for cardiac treatment.

## 3. NDDS Overcoming OIADD Limitations

For OIADD, the key problems to be solved by the NDDS may vary depending on the disease characteristics, target sites, and drug properties. For example, when treating diseases localized in the lungs, the primary challenge is to maximize drug retention and avoid clearance. In diseases characterized by thickened pulmonary mucus layers, the critical issue is overcoming the mucus barrier to deliver the drug to the target site. For drugs with significant systemic toxicity that require long-term administration or large doses, improving targeting, modulating release, and reducing dosage are the primary concerns. The development of NDDS brings hope to overcome the limitations of OIADDs in a wide range of diseases, presenting the potential to overcome barriers, prolong retention time, modulate release, and realize targeted effects. The mechanisms for realizing these functions and the research progress are described below.

### 3.1. Overcoming Barriers

The upper and lower respiratory tracts have similar intrinsic physiological barriers and cell type characteristics. Overcoming these barriers primarily involves addressing the mucus and cell membrane barriers. The mucus barrier extends throughout the respiratory tract, where mucin is secreted by goblet cells in the nasal and respiratory tracts, along with secretions from the mucous glands and cilia, forming the mucociliary clearance (MCC) system. This system effectively captures a large number of molecules and transports them either to the digestive system or out of the body via coughing or swallowing [47,48]. To ensure effective drug delivery, NDDS must penetrate the cell membrane barrier after overcoming the mucus barrier.

#### 3.1.1. Penetrating the Mucus Barrier

The study of NDDS strategies for overcoming the mucus barrier is crucial for treating diseases characterized by thickened mucus layers, such as COPD, asthma, and cystic fibrosis. Two primary approaches have been employed to effectively overcome this barrier: mucus-adherent NDDS and mucus-penetrating NDDS. However, the mucus layers in the nasal cavity and lungs are continuously secreted and shed via various clearance mechanisms. In healthy individuals, the nasal mucus layer is renewed every 20~30 min [49], while the airway mucus layer is refreshed approximately every 20 min [50]. Because of this rapid renewal process, mucus-adherent NDDS are cleared over time, making it challenging for them to reach the epithelial cells in the submucosal layer. Furthermore, studies have shown that mucus-penetrating nanoparticles are widely distributed throughout the airways of mice after inhalation, whereas mucus-adherent nanoparticles are not as effective [51]. Consequently, a mucus-penetrating NDDS strategy is often considered the superior choice. The following sections focus on this type of NDDS.

Surface properties are critical factors that influence penetration of the NDDS mucus layer. Because mucin fibers in mucus can form adhesive interactions with hydrophobic regions on the surface of foreign particles, hydrophilic substances usually facilitate the penetration of mucus [52]. Numerous studies have shown that some hydrophilic or amphiphilic neutrally charged polymers, such as poly(2-alkyl-2-oxazoline) [53], polydopamine poly (2-alkyl-2-oxazolines) [54], poly(vinyl alcohol) [55], and poloxamers [56], enhance the ability of NDDS to penetrate mucus. The frequent use of PEG for chemical conjugation or physical adsorption on nanoparticles to facilitate mucus penetration can be attributed to its neutral charge and hydrophilic surface, which help it to “hide” during mucus penetration, thereby improving its effectiveness [57,58]. Wu et al. [59] developed an NDDS that is capable of penetrating the mucus barrier in the lungs by combining hydrophilic hyaluronic acid with PLGA containing polymyxin B in a water–oil system, yielding produce nanoparticles with good hydrophilicity. The nanoparticles showed more than 2-fold mucus penetration efficiency in vitro compared to free polymyxin B. The nanoparticles had a good hydrophilicity. In addition, the surface charge of NDDS is one of the factors affecting mucus penetration. Li et al. [60] introduced ICG-labeled liposomes into a transwell artificial mucus model and measured the concentration of liposomes that penetrated the mucus at various time points (1, 6, 12, and 24 h). They found that neutral liposomes penetrated the mucus at rates approximately 1.5 to 2.5 times higher than those of negatively charged liposomes and 2~4 times higher than those of positively charged liposomes. Laffleur et al. [61] reported neutral nanoparticles composed of polyacrylic acid (PAA) and polyallylamine (PAM), whose diffusion efficiencies in the mucus layer were 1.8 and 2.5 times higher than those of negatively charged PAA and positively charged PAM nanoparticles, respectively. These studies suggest that the surface neutrality of NDDS may result in superior penetration into mucus.

**Figure 1:**
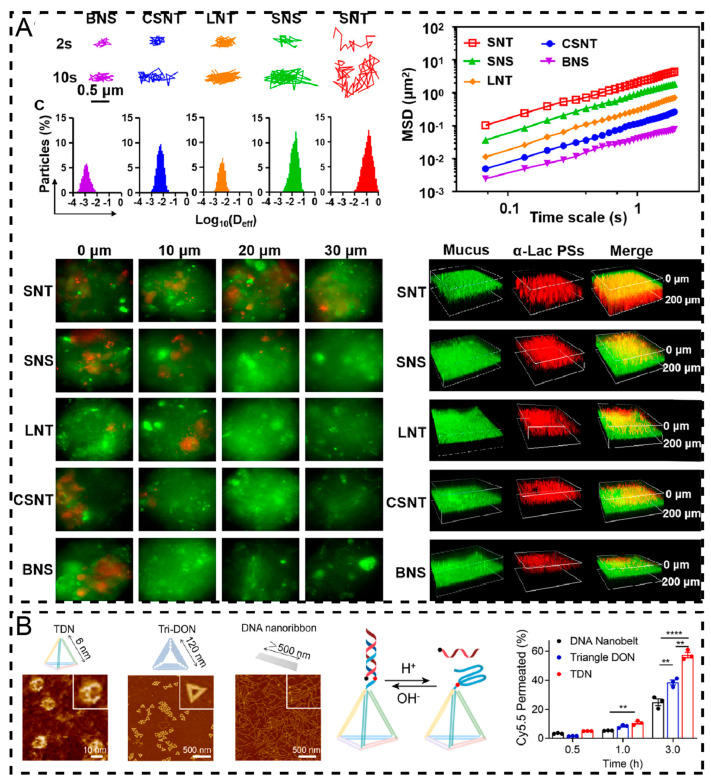
Aerosolized NDDS overcomes different physiological barriers. (**A**) Representative trajectories of α-lactalbumin-derived peptides in intestinal mucus for 2 and 10 s were observed using multiparticle tracking. The logarithmic distribution of the effective diffusion coefficient of individual particles over 1 s is demonstrated. In ex vivo experiments, Cy5-labeled α-lactalbumin-derived peptides (red) were co-incubated with Alexa Fluor 488-WGA-labeled intestinal mucus (green) to visualize the two-dimensional diffusive coverage of the peptides in the mucus. Three-dimensional distributional imaging after 3 h of permeabilization demonstrates the distribution within a depth of 0–30 μm. Adapted from ref. [63] with permission. (**B**) Schematic representation of DNA tetrahedrons, triangle DNA origami nanostructures, and DNA nanoribbons dissociated into i-primary structures upon acidic stimulation (**: *p* < 0.01, ****: *p* < 0.0001). Adapted from ref. [64] with permission.

Size and shape are crucial properties that influence the ability of NDDS to penetrate the mucus layer. Schuster et al. [62] found that PEGylated nanoparticles with diameters of 100 nm and 200 nm rapidly penetrated respiratory mucus, while those with diameters ≥500 nm were immobilized by the mucus mesh. Bao et al. [63] prepared α-lactalbumin polypeptidomes of different shapes and sizes, including large spherical (200~300 nm in diameter), small spherical (20~30 nm in diameter), long tubular (800~1200 nm in length), and short tubular (100~200 nm in length), and observed the fluorescence intensities of Cy5-labeled α-lactalbumin polypeptide particles at mucus depths of 10, 20, and 30 μm (Figure 1A). They found that the short tubular α-lactalbumin polypeptidomes were widely dispersed in the mucus at a depth of 30 μm after 3 h of infiltration in the mucus, the small spherical and long tubular α-lactalbumin polypeptidomes were able to penetrate to the depths of 20 μm and 10 μm, respectively, while the large spherical α-lactalbumin polypeptidomes stayed on the surface of the mucus and were not able to penetrate into the mucus layer effectively. Researchers have also explored other interesting nanocarrier shapes. For example, Fan et al. [64] prepared inhalable nanocarrier framework nucleic acids with three structures (DNA tetrahedrons, triangular DNA origami nanostructures, and DNA nanoribbons) for the treatment of metastatic lung cancer by mixing a specific amount of single DNA strands in an appropriate buffer, utilizing precise temperature control to ensure that the DNA strands were correctly folded into the preset structures, and evaluated their transmucosal ability (Figure 1B). The results showed that DNA tetrahedrons had superior mucus penetration, with permeation rates approximately 1.5 and 2.5 times higher than those of the triangle DNA origami structures and DNA nanoribbons, respectively [64]. This study offers valuable insights into the design of precise delivery systems.

#### 3.1.2. Penetration of Cell Membrane Barriers

Pulmonary surfactants (PS), synthesized and secreted by type II alveolar epithelial cells (AECs II), are primarily composed of 92% dipalmitoylphosphatidylcholine (DPPC) and 8% surfactant proteins (SP-A to SP-D), whose main function is to maintain the balance of alveolar surface tension to prevent alveolar collapse [65,66]. Lipid-based NPs (LNPs) are commonly used to achieve cellular internalization and overcome the cell membrane barrier because of their similarity in composition to PS [66,67]. Wang et al. [68] reported a liposome (PSB) consisting of DPPC/POPG/DPPG/CHO (20:9:5:4) supplemented with Ca^2+^ for the inhalation delivery of astaxanthin and pirfenidone for the treatment of idiopathic pulmonary fibrosis (IPF). Transmission electron microscopy showed that liposomes with added Ca^2+^ had a multilayered morphology, consistent with the typical PS micromorphology, which enhanced the absorption potential of PSB in the lungs. Compared to conventional PEG liposomes and cationic liposomes, PSB liposomes showed a 2-fold improvement in uptake by mouse alveolar epithelial type II (AECs II). Moreover, the clinical success of Arikayce^®^, the only FDA-approved inhaled nanoparticle formulation, which is a cholesterol- and DPPC-based amikacin liposome suspension for the treatment of Mycobacterium avium complex lung disease, further underscores the clinical translational potential of LNPs [69].

Cell-penetrating peptides (CPPs), which facilitate cellular entry by transiently disrupting cell membranes, represent another widely used strategy to overcome cell membrane barriers [70]. Conjugating CPPs with nanoparticles can significantly improve cell membrane penetration efficiency [71,72]. For example, the TAT peptide, a typical CPP composed of 11 amino acids, has been shown to effectively enhance the cellular internalization of NDDS [73]. Kleemann et al. [74] developed a TAT-PEG-PEI conjugate by covalently linking an oligopeptide associated with the protein transduction domain of HIV-1 TAT to PEI using PEG as a linker, forming a nanoscale polyplex after binding with DNA. In vivo transfection experiments showed that the transfection efficiency of this polyplex was six-fold higher than that of DNA conjugated to PEI without TAT modification.

#### 3.1.3. Crossing Multiple Barriers

As previously mentioned, PEGylation is a common approach for endowing NDDS with mucus-penetrating capabilities. However, some studies have raised concerns about this modification. Research has indicated that PEGylated NPs exhibit significantly reduced uptake in Calu-3 cells compared with unmodified NPs [75]. This suggests that a single material may struggle to achieve excellent mucus penetration and cell membrane permeability simultaneously. Consequently, employing a multicomponent system may be an effective strategy for enhancing mucus permeability and cell membrane penetration. Dixon et al. [76] utilized a novel cell-penetrating peptide, glycosaminoglycan-enhanced transduction peptide (GET peptide), which was first conjugated with a 5 kDa PEG chain via a maleimide–thiol coupling reaction and then directly complexed with DNA through electrostatic interactions to create a peptide–polymer hybrid nanoparticle. These nanoparticles were designed to enhance mucus and cell membrane permeability for DNA delivery. The screening results showed that nanoparticles containing 40% PEG exhibited transgene efficiency approximately five times higher than that of pure DNA. Leal et al. [77] constructed a phage library, screened it to obtain peptides with mucus-penetrating ability, and covalently bound them to carboxylic acids on modified polystyrene nanoparticles to enhance their mucus-penetrating properties. The peptide-modified nanoparticles diffused approximately 600-fold faster in sputum from cystic fibrosis patients than in positively charged control phages. In addition, the cellular uptake of the nanoparticles was improved by 1.6- to 2.2-fold compared to mPEG 1kDa conjugated nanoparticles with the same mucus penetration.

In addition to the mucus and cell membrane barriers, other specific impediments may exist in different diseases. IPF is an irreversible and fatal form of interstitial fibrosis. Nidanib is an FDA-approved first-line oral agent; however, its low bioavailability and limited lung targeting necessitate the use of high doses, potentially causing hepatorenal toxicity. In contrast, navitoclax (ABT-263) can remove senescent epithelial cells without damaging the normal cells. Yang et al. [78] developed liposomes (AN-TR) modified with tri-(2-carboxyethyl)phosphine (TCEP) and l-arginine to co-deliver these two drugs. TCEP promotes mucus penetration through charge repulsion and a chemical bond-breaking mechanism, whereas L-arginine promotes NO production and activates matrix metalloproteinases (MMPs) to degrade the ECM, thereby enhancing the efficiency of targeted delivery to deep alveoli. To construct a dual-barrier model containing a tracheal mucus layer and a fibroblast collagen layer, a mucus layer was added to the BEAS-2b cell layer placed in the Transwell chamber, and a collagen layer was added to the fibroblast layer on the culture plate to simulate the ECM environment in the alveolar interstitium. Subsequently, they labeled the liposomes with 1,1′-dioctadecyl-3,3,3′,3′-tetramethylindocarbocyanine perchlorate (DiI) to evaluate their ability to penetrate the dual barriers. The experimental results showed that the cellular uptake of AN-TR was approximately twice that of liposomes modified with TCEP or L-arginine alone, demonstrating the effectiveness of the combined TCEP and L-arginine modification strategy in overcoming the dual barriers in IPF.

### 3.2. Prolonged Retention

Generally, drugs absorbed through the nose or lungs can rapidly enter the systemic circulation; however, for the treatment of pulmonary and nasal diseases, it is desirable for NDDS to achieve prolonged drug residence at the target site. NDDS can extend local residence time and enhance drug efficacy by avoiding clearance, modulating interactions with extracellular barriers, and modulating carrier properties, among other strategies.

#### 3.2.1. Avoidance of Clearance

There are two main natural clearance mechanisms in the respiratory tract: MCC and immune clearance. Penetration of the mucus barrier, as mentioned previously, is not only a way to reach the treatment site but also a strategy to avoid the natural clearance of MCC from the respiratory tract. This section describes how inhaled drugs can avoid immune clearance through the NDDS.

**Figure 2:**
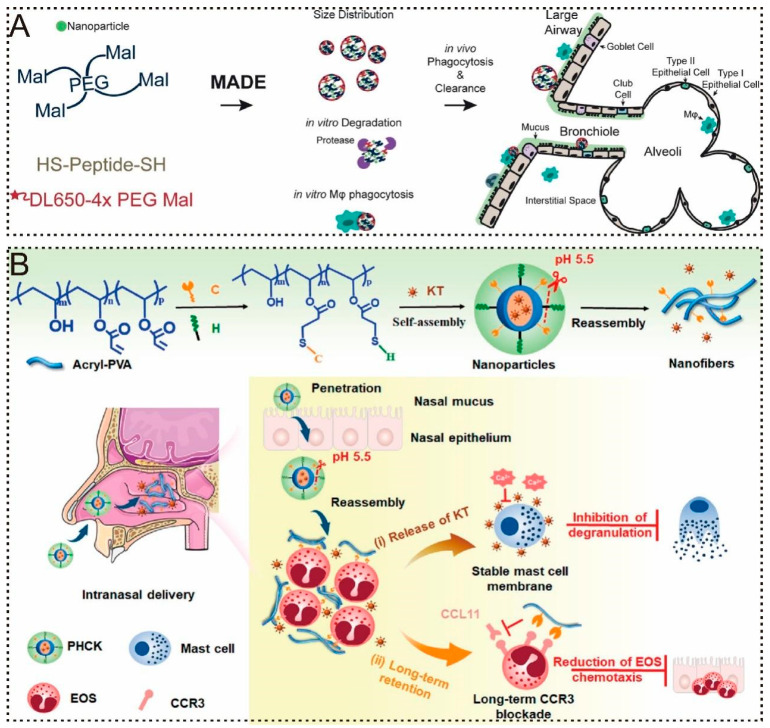
Strategies for providing extended retention of atomized NDDS. (**A**) Schematic diagram of the N-in-M system demonstrating the structure of the N-in-M system and showing the mechanism of nanoparticle release. Adapted from ref. [83] with permission. (**B**) Synthesis route of PHCK and the principle of prolonged retention time and treatment of allergic rhinitis. Adapted from ref. [84] with permission.

As a key component of the immune system, macrophages are responsible for phagocytosis and removal of invading microorganisms and particulate matter and are present throughout almost the entire respiratory tract, especially alveolar macrophages, which are the core cell type of the local immune response in the lungs [79,80]. Aerosolized drugs exhibit different geometric particle sizes ($D_{v}$), shapes, densities, porosities, and agglomeration structures. In order to describe the behavior of different particles in a fluid in a uniform way, the concept of aerodynamic diameter of nebulization ($D_{ae}$) is introduced, which is the diameter of a sphere of unit density with the same settling velocity as the particles. The relationship between $D_{v}$ and $D_{ae}$ can be expressed as follows:

$$D_{ae}=D_{v}\sqrt{\frac{\rho }{{\chi \rho }_{0}}}$$

where χ is the dynamic shape factor, ρ is the density of spherical particles, and $\rho_{0}$ is the unit density, usually expressed as $\rho_{0}$ = 1.00 g/cm^3^). The aerosol particle size distribution often follows a lognormal distribution and is characterized by the aerodynamic mass median particle size (MMAD), which indicates whether 50% of the atomized particles are larger or smaller than the $D_{ae}$ value for a specified size. For macrophages, whose phagocytosis of nanoparticles is influenced by the size and elasticity of the particles, it readily phagocytizes particles with $D_{v}$ in the range of 1~5 μm [81], as well as stiffer particles due to the high elastic modulus of these particles, which limits their deformability and makes them more susceptible to encapsulation and phagocytosis upon contact with macrophages [82]. Once phagocytosed, these particles are transferred to the respiratory mucosa for clearance. Mejías et al. [83] developed a novel nanomicrogel system (N-in-M) that offers a new strategy for the treatment of respiratory diseases by reducing macrophage phagocytosis through the modulation of the N-in-M size and elastic modulus by controlling the concentration of polymers. This system encapsulates drug-loaded nanoparticles in a solution containing four-arm PEG-maleimide and a neutrophil elastase-responsive peptide, which undergoes Michael addition crosslinking to form N-in-M-embedded nanoparticles (Figure 2A). N-in-M has an MMAD of 1~5 μm, which makes it suitable for deposition deep in the lungs, and its $D_{v}$ was controlled between 4 and 8 μm, which is beyond the size that is readily phagocytosed by macrophages. When N-in-M encounters overexpressed proteases in an inflamed pulmonary environment, the gel network rapidly responds and degrades, releasing nanoparticles. The phagocytic rate of macrophages treated with N-in-M at a polymer concentration of 50% *w*/*v* in the gel was approximately 3-fold lower than that of macrophages treated with N-in-M at a polymer concentration of 20% *w*/*v*. This is because increasing the gel concentration within the N-in-M results in a larger size and reduced elasticity, both of which contribute to the avoidance of phagocytosis by the macrophages. Furthermore, Teng et al. [84] selected PVA as a backbone molecule (P) and grafted pH-cleavable hydrophilic terminal mPEG2000-Hyd-SH (H) and CCR3 antagonist peptide (C), which inhibit eosinophil (EOS) chemotaxis, to prepare ketotifen (KT) nanoparticles (PHCK) for the treatment of allergic rhinitis (Figure 2B). The nanoparticles are responsive to the breakage of PEG in PHCK in a weakly acidic environment suffering from rhinitis, allowing the nanoparticles to be converted into nanofibers, which can then cover the cell surface and reduce phagocytosis by macrophages. Microscopic scans showed spherical particles on the surface of cells treated with PCK without responsive PEG grafting in PBS (pH 5.5) solution; PCK was mainly distributed inside the cells, whereas a network structure formed by entanglement of nanofibers was observed on the surface of cells treated with PHCK, and PHCK was mainly enriched around the cell membrane. Further in vivo distribution observations revealed that in the nasal cavity of the allergic rhinitis rat model, the fluorescence intensity of the PCK group was significantly reduced at 12 h, whereas the retention time in the nasal mucosa of the PHCK group was extended to 24 h. These results demonstrate that a change in the shape of the drug carriers could affect the retention time.

Cell membrane cloaking is a novel approach aimed at enhancing therapeutic efficacy by evading clearance by the immune system. Chen et al. [85] utilized this strategy to prepare desferrioxamine-containing PLGA nanoparticles using macrophage membranes as the shell, which showed an approximately 2.5-fold reduction in macrophage uptake compared to nanoparticles not encapsulated with macrophage membranes, thus allowing for prolonged nanoparticle retention time in the lungs and improved efficacy in the treatment of bronchopulmonary dysplasia. Suitable genetic engineering techniques to modify cell membranes could further enhance their ability to avoid immune phagocytosis compared to purely naturally derived cell membranes. Proline–alanine–serine (PAS) is a hydrophilic polypeptide chain that adds PAS to proteins or NDDS through genetic engineering to take advantage of its hydrophilicity and electroneutrality to avoid in vivo clearance and prolong circulation time [86]. Duan et al. [87] transfected THP-1 cells with a fusion protein plasmid containing Igk, a signaling sequence that directs the translocation of proteins to the cell surface, a transmembrane anchor structural domain of the cell membrane surface receptor PDGFR, and fluorescent protein-tagged PAS via a lentivirus to express PAS on the cell surface. They obtained genetically engineered modified cell membranes and natural macrophage membranes by cell lysis and differential centrifugation and then coated the membranes onto PLGA nanoparticles by sonication to form genetically engineered modified cell membrane-coated nanoparticles (PAS-MΦ-NPs) and natural-type macrophage membrane-coated nanoparticles (WT-MΦ-NPs). In both in vitro experiments and an LPS-induced pneumonia mouse model, the macrophage uptake of PAS-MΦ-NPs was significantly lower than that of WT-MΦ-NPs, demonstrating enhanced immune evasion capabilities.

#### 3.2.2. Modulating Interactions with the Extracellular Barrier

Increased mucosal adhesion can prolong local retention time [88,89]. In one study, researchers prepared gel solutions containing nanolipid carriers (NLC) and nanoemulsions capable of adhering to mucins [90] The solution had a sol–gel transition temperature of 28~37 °C and could form an in situ gel after spraying into the nasal cavity to improve the retention time of the drug in the nasal cavity. The presence of lipid-based nanosystems increased the viscosity of the gel and prolonged the retention time compared to gels that did not contain NLC and nanoemulsions.

Another strategy for prolonging drug retention is to modify the carrier structure [91]. The new coronavirus SARS-CoV-2 infects host cells by binding to the angiotensin-converting enzyme (ACE) II receptor, which is highly expressed in airway epithelial cells. In one study, researchers designed an aerosolized inhalation of raltegravir nanoemulsion (RDSV-NE-AYQ) that specifically binds to ACE II receptor-binding peptide modifications [92] and quantified the retention time of raltegravir and its metabolites in the lung tissue. After 3 h of administration, the lung concentration of RDSV-NE-AYQ was 2.3-fold higher than that of the unmodified ACE II receptor-binding peptide nano-emulsion, suggesting that modification of the ACE II receptor-binding peptide significantly prolonged the nanoemulsion’s retention time in the lung tissue.

#### 3.2.3. Modulating Carrier Properties

In addition, penetration into the bloodstream can be reduced, and the retention time of the drug in the body can be prolonged by adjusting the size of the drug carrier. Ryan et al. [93] found that the systemic absorption of polyethylene glycolized dendritic polymers was negatively correlated with molecular weight and that the retention time in the lungs was positively correlated with molecular weight over a range of sizes. The relatively small (<22 kDa) dendritic polymers showed a systemic absorption of approximately 20–30% and almost disappeared from the lungs after 48 h. The larger (78 kDa) polyethylene glycolized dendritic polymers showed an absorption of only 2% and a retention time of up to seven days in the lungs. The molecular weight of dendritic polymers can be increased for NDDS that wish to be retained locally, whereas the molecular weight of dendritic polymers can be decreased for NDDS that wish to enter the cardiovascular system.

### 3.3. Modulated Release

The modulated release includes both temporal and spatial dimensions. Spatially, the drug is released at a specific location by the carrier in response to physicochemical stimuli of the pathological environment. Temporally, it includes slow-controlled, sequential, and rhythmic releases. Research on rhythmic drug release systems has emphasized the importance of developing drug release strategies that synchronize with human physiological rhythms. For example, the oral Pulsincap^®^ and implantable Port^®^ systems utilize mechanisms such as swelling, erosion, osmosis, and rupturable coatings to achieve timed and precise drug release, which is particularly suitable for diseases such as diabetes and hypertension that require medication to be taken at specific intervals to coincide with the physiological cycle, thereby improving therapeutic efficacy and patient adherence [94]. Although there are no relevant studies on transnasal or transoral nebulized pulsatile drug release systems, in-depth studies on brain rhythms (cerebrospinal fluid dynamics, brain waves, etc.), respiratory rhythms (respiratory rate, respiratory depth, respiratory pattern, etc.), cardiovascular rhythms (heart rate, blood pressure, blood flow, etc.), and other fields, and aerosolized rhythmic drug release systems are expected to become an important direction of development in the future.

#### 3.3.1. Physicochemical Stimulus-Responsive Release

The targeting and regulatory drug release behaviors of physicochemical stimulus-responsive NDDS are often inextricably linked. Physicochemical stimulus-responsive NDDS are sensitive to specific physicochemical properties in the environment and can enrich target tissues or cells for the targeted delivery and release of drugs in response to specific environmental conditions [95]. The precision and efficiency of the treatment can be improved by triggering drug release in response to specific conditions. Compared to systemic delivery, the need for the targeted action of NDDS is still significant in OIADD, although the need for its modulated release action is reduced in some cases.

The physicochemical stimulus responsiveness of aerosolized NDDS includes sensitivity to both intrinsic (e.g., pH, redox state, enzyme activity, and ion concentration) and extrinsic (e.g., temperature, light, ultrasound, magnetic, and electric fields) stimuli. The main types of nanodelivery carriers are lipid-based, polymeric, inorganic, and biomimetic NDDS. The majority of external stimuli-responsive NDDS is intricately linked to the utilization of inorganic materials, particularly metals [96]. However, the potential localized toxicity of inorganic nanocarriers cannot be overlooked [58]. For instance, extensive exposure of the lungs to inorganic nanocarriers may induce severe inflammation and oxidative stress, whereas their accumulation in vivo can result in severe cardiotoxicity and neurotoxicity [97]. Consequently, current research on physicochemical stimuli-responsive NDDS for aerosol inhalation increasingly favors the use of materials with superior biocompatibility and biodegradability.

**pH Response.** In the pathological environment of the lungs, nose, brain, heart, and other related diseases, such as areas of inflammation, tumor microenvironments, and ischemic tissues, acidic metabolic wastes typically accumulate owing to high metabolic activity and inadequate perfusion, giving these environments an acidic character. These acidic conditions provide a basis for the application of pH-responsive drug delivery systems [98,99,100]. For instance, when the environmental pH decreases, a DNA sequence rich in cytosine bases forms stable C-C+ base pairs, leading to protonation and formation of a four-stranded i-motif structure, which is highly stable under acidic conditions. [101]. Fan et al. [64] utilized the acid-responsive properties of the i-motif to develop an inhalable pH-sensitive tetrahedral DNA carrier loaded with aptamers for the treatment of metastatic lung cancer. Fluorescence quenching studies revealed that when the pH of the environment decreased from 7.4 to 6.5, the cytosine bases in the DNA carrier formed an i-motif structure, resulting in the release of the encapsulated aptamers, as evidenced by an increase in fluorescence intensity. Conversely, when the pH was reverted from 6.5 to 7.4, the fluorescence intensity diminished, confirming the capability of this pH-responsive DNA carrier to selectively release therapeutic agents in the acidic microenvironment of tumors.

**Redox response.** The Redox response is an important biological process that plays a key role in cell signaling. Glutathione (GSH) is a typical biomarker of the reduction response, and its concentration in the tumor cytoplasm is approximately four times that in normal cells [102]. Because of its ability to cleave disulfide and diselenide bonds, which are susceptible to reduction reactions, GSH is often used as an ideal choice for reduction-responsive drug release [103]. Reactive oxygen species (ROS) is a general term for a class of oxidative response biomarkers, including H_2_O_2_, O_2_^−^, and ^●^OH and their high reactivity triggers oxidative stress, which is closely associated with a variety of diseases [104], including most brain diseases, IPF, and ARDS [18,45,105,106,107]. One strategy to achieve drug release at the lesion site is to utilize chemical bonds that are susceptible to oxidation. The introduction of redox-sensitive chemicals into NDDS to trigger the response mechanism can achieve drug release at the lesion site. For example, Liu et al. [108] prepared olanzapine nanoparticles by conjugating a phenylboronic pinacol ester with dextran for intranasal administration for the treatment of depression. These nanoparticles utilize an ROS-sensitive boronate ester bond to specifically release encapsulated olanzapine and scavenge the depressed brain. They measured the nanoparticle size in the presence and absence of H_2_O_2_ and found that the particle size significantly decreased in the presence of H_2_O_2_, indicating the successful degradation of the nanoparticles. ROS-responsive release experiments conducted using the dialysis method showed cumulative release rates of 65.13% and 93.38% after 8 h in the presence and absence of H_2_O_2_, respectively.

**Figure 3:**
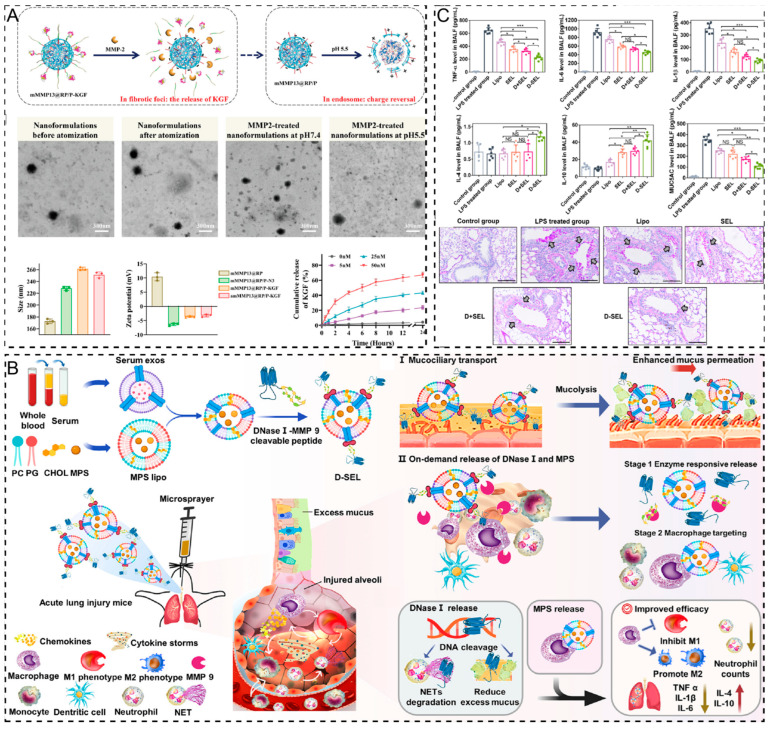
The ability of atomized NDDS to modulate drug release. (**A**) Schematic representation of the responsiveness and pH-sensitive mechanism of mMMP13@RP/P-KGF to MMP-2 and transmission electron microscopy (TEM) images of mMMP13@RP/P-KGF, amMMP13@RP/P-KGF and their nanoparticles pretreated with MMP-2 at pH 7.4 or 5.5 conditions are shown, scale bar: 300 nm. The particle sizes and zeta potentials of mMMP13@RP, mMMP13@RP/P-N3, mMMP13@RP/P-KGF, and amMMP13@RP/P-KGF were measured by dynamic light scattering, and the cumulative releases of KGF from amMMP13@RP/P-KGF were also demonstrated after treatment with different concentrations of MMP-2. Error lines indicate standard deviation (n = 3). Adapted from ref. [110] with permission. (**B**) Schematic diagram of the MPS/D-SEL system demonstrating its mucus penetration and sequential drug release mechanism. (**C**) After inhalation of MPS-loaded lipo, SEL, D-SEL, or the physical mixture of MPS/SEL and free DNase I, the levels of anti-inflammatory cytokines (IL-4 and IL-10) in BALF, the level of MUC5AC in BALF, AB-PAS staining images showing goblet cell metaplasia (scale bar = 50 μm), and H&E staining images (scale bar = 100 μm; zoom-in images scale bar = 50 μm; * *p* < 0.05, ** *p* < 0.01, *** *p* < 0.001, NS: *p* > 0.05). Adapted from ref. [113] with permission.

**Enzyme Response.** Abnormal expression of certain enzymes under pathological conditions can trigger enzyme response mechanisms to achieve targeted drug release [109]. MMPs are able to catabolize the ECM and play a key role in various physiological and pathological processes, such as tissue remodeling, healing, tumor invasion, metastasis, and fibrosis. Zhang et al. [110] developed a ribosomal protein-loaded mMMP13-core nanoparticle and engineered a dual-drug-loaded formulation, amMMP13@RP/P-KGF, by conjugating keratinocyte growth factor (KGF) to the nanoparticle via a matrix metalloproteinase 2 (MMP-2)-responsive peptide. In the lung fibrosis environment, MMP-2 hydrolyzes the responsive peptide, releasing KGF from the nanoparticle surface and exposing the RGD motifs of ribosomal proteins. This mechanism allows the specific delivery of mMMP13 to target cells. ELISA-monitored KGF release profiles showed that after 6 h of incubation with 50 × 10^−9^ M MMP-2, amMMP13@RP/P-KGF released approximately 50.1% of KGF, which was approximately 21 times higher than the release observed in the MMP-2-free group (Figure 3A). MMPs are essentially gelatinases [111]. Gou et al. [112] engineered a gelatin–silk protein composite particle (GSC) that exhibited pronounced responsiveness to high MMP-9 expression in the tumor environment. After incubation with MMP-9-containing buffer, there was no significant difference in the drug release rate between MMP-9- and PBS-incubated GSCs for the first 30 min. However, when the incubation time was longer than 30 min, the drug release rate of GSCs incubated with MMP-9 increased significantly and reached a peak after 80 min of incubation. This delayed-release profile suggests that GSCs effectively concentrate at tumor sites, thereby enabling precise drug delivery.

#### 3.3.2. Sustained Release and Controlled Release

In OIADD, sudden exposure of nasal or pulmonary cells to high concentrations of certain drugs can compromise delicate respiratory structures, leading to severe local inflammation and other adverse effects [114]. Moreover, while drugs absorbed through the nasal or pulmonary routes can rapidly enter the systemic circulation in significant amounts, this can also result in systemic side effects. Sustained-release and controlled-release NDDS in inhalation therapies not only reduce dosing frequency and enhance patient compliance but also mitigate toxic side effects by gradually and precisely releasing the drug in targeted regions, thereby avoiding abrupt local or systemic exposure.

Although microspheres are not strictly nanosized, they are commonly used as carriers for slow- and controlled-release systems. For example, large porous PLGA microspheres have demonstrated a wide range of potential applications in the pulmonary delivery of hydrophobic drugs, ensuring a favorable safety profile and a modifiable sustained effect. Ondansetron is a selective 5-HT3 receptor antagonist used to prevent nausea and vomiting induced by chemotherapy, radiotherapy, and postoperative vomiting. In a pharmacokinetic study conducted by Gungor et al. [115], ondansetron-loaded PLGA microspheres were administered intranasally to rats, and plasma ondansetron levels were maintained within the range of 30–48 ng/mL for over 96 h, demonstrating sustained drug delivery behavior and relatively constant plasma drug concentrations. In addition to conventional microsphere carriers, novel polymeric nanodelivery systems such as nanosponges are considered superior carriers because of their high porosity and programmable release capabilities [116,117]. Polymeric nanofibers fabricated via electrospinning have shown tremendous potential owing to their high loading capacity and tunable drug release rates achieved by modifying their composition and structure [118,119,120]. The emergence of these innovative carriers offers new perspectives for the design of controlled-release systems within OIADD.

Despite the numerous advantages of NDDSs, not all drugs are suitable for formulation into sustained- or controlled-release NDDS, particularly OIADD. For instance, drugs requiring high doses or those with poor solubility, which result in low drug loading in aerosolized NDDS, may pose a risk of damaging delicate pulmonary and nasal tissues because of the large quantities of drugs and excipients stored within the NDDS. Moreover, traditional strategies to increase drug dosage by expanding the administration volume can prolong the aerosolization time, thereby diminishing patient compliance. Therefore, when formulating drugs into aerosolized sustained-release or controlled-release NDDS, it is imperative to carefully consider the physicochemical properties of the drug, the required dosage, and the therapeutic needs of the target disease.

#### 3.3.3. Sequential Release

Monotherapies often yield suboptimal results in diseases with complex pathological mechanisms. In such cases, a multidrug sequential release system may enhance patient compliance and deliver superior therapeutic outcomes compared to conventional drug delivery approaches [121]. For instance, in acute lung injury, neutrophil extracellular traps (NETs) are closely associated with a mucus-obstructed pulmonary microenvironment, leading to the hyperactivation of alveolar macrophages. M1 macrophages, in turn, drive further neutrophil infiltration into the lungs, perpetuating NET formation and exacerbating alveolar damage. To address this, Liu et al. [113] designed an inhalable nanocarrier, MPS/D-SEL, which consisted of a serum exosome and liposome hybrid system (SEL) internally encapsulated with methylprednisolone sodium succinate (MPS) and externally chemically coupled with DNase I via the degradable enzyme MMP-9 to achieve dual drug delivery. First, this dual-drug delivery system releases DNase I at the site of injured alveoli, degrades NETs, and mitigates the inflammatory microenvironment, thereby preventing the further activation of M1 macrophages. The degradation of MMP-9 allowed for the controlled release of MPS, promoting the polarization of macrophages from the M1 to M2 phenotype, thereby suppressing inflammation, fostering tissue repair, and further consolidating the anti-inflammatory effect (Figure 3B). Analysis of the levels of inflammatory factors in the BALF of mice with acute lung injury showed that the sequential release system decreased the levels of pro-inflammatory cytokines by approximately 1.5-fold and increased the levels of anti-inflammatory cytokines by approximately 1.3–1.7-fold compared to the physical mixture of MPS-SEL and DNase I, suggesting that MPS/D-SEL controls DNase I and MPS in temporally controlled release, confirming that MPS/D-SEL is effective in attenuating excessive inflammation induced by LPS in mice (Figure 3C).

### 3.4. Targeted Action

The NDDS is a key tool for modulating drug-targeting behavior. In general, the design of a targeted NDDS for systemic administration requires directing the drug to a specific organ and further targeting specific cells or organelles. This necessitates the consideration of additional factors in target design. Traditional routes of drug delivery often have insufficient drug accumulation in the target organ; for example, intravenous injection suffers from drug leakage and shear forces generated by blood flow, in addition to drug leakage before the NDDS reaches the target site [122], which must also be cleared by the liver and spleen during blood circulation [123]. By contrast, orally administered drugs must overcome metabolism in the gastrointestinal tract and traverse the gastrointestinal mucosa before entering the body. In contrast, OIADD can deliver drugs directly to the lungs and nose or bypass the BBB after nasal nebulization and deliver drugs directly to the brain, which has a “fast-track” approach has a “passive targeting” effect, greatly reducing the difficulty in designing and translating NDDS for these diseases. This “fast track” itself contains a “passive targeting” effect, which greatly reduces the difficulty of designing and translating NDDS for these diseases.

The main targeting strategies for NDDS in OIADD include stimuli-responsive and ligand-receptor binding targeting. Because stimuli-responsive targeting is often closely related to the drug release process, and most stimuli-responsive NDDS in OIADDs are more focused on modulating drug release through stimulus-response, they are presented in the modulation of drug release section of this paper. Ligand-receptor binding targeting relies on the binding of ligands (e.g., antibodies, peptides, small molecules) in the carrier to specific or highly expressed receptors in the pathological environment, which triggers phagocytosis or endocytosis of the nanocarrier, thus realizing the precise delivery of the drug [124]. Table 2 shows several examples of OIADD actively targeting NDDS receptors, preparation methods, and application effects in therapeutic drugs for lung and brain diseases.

**Figure 4:**
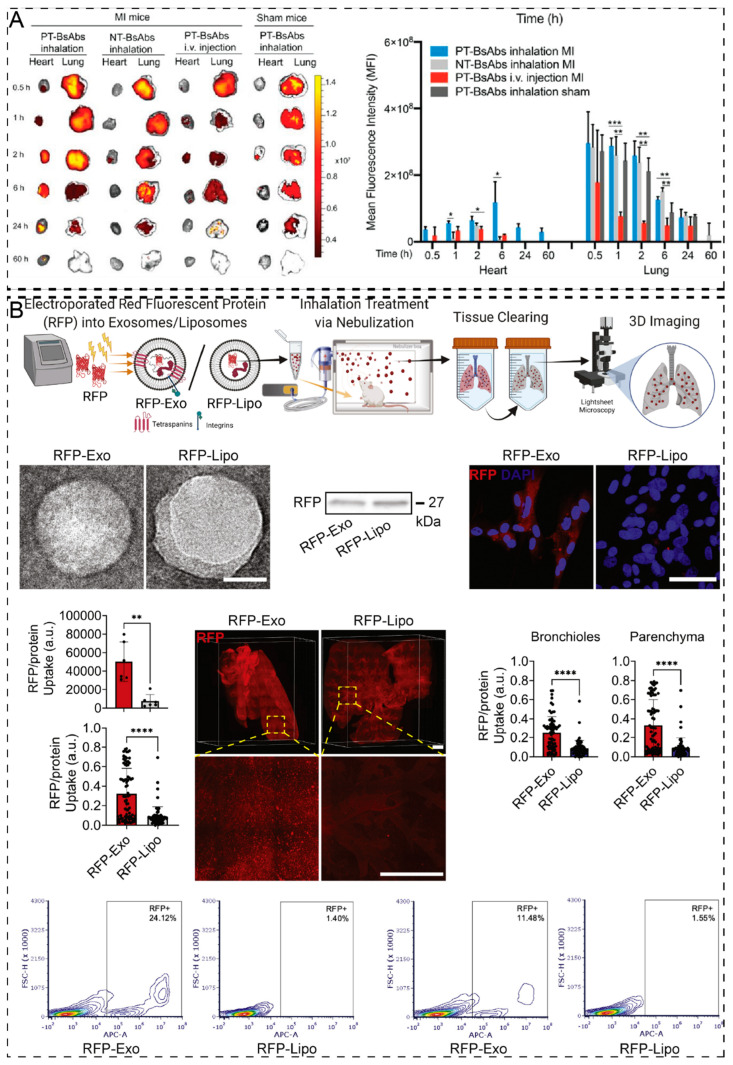
Targeting ability of aerosolized NDDS. (**A**) Biodistribution of inhaled PT-BsAb assessed by fluorescence imaging (*: *p* < 0.05, **: *p* < 0.01, ***: *p* < 0.001). Adapted from ref. [125] with permission. (**B**) Preparation and distribution of exosomes and liposomes (**: *p* < 0.01, ****: *p* < 0.0001); scale bar: 50 nm, 50 μm and 1000 μm. Adapted from ref. [139] with permission.

Additionally, aerosol therapy for the same NDDS may be more targeted than systemic administration for the treatment of other diseases. Liu et al. [125] designed a bispecific antibody (PT-BsAb) that targeted both hematopoietic stem cells (HSCs) via CD34 and platelets via CD42b to redirect lung-resident HSCs to the injured heart after acute myocardial infarction. Upon administration, it binds to HSCs and platelets in the lungs. By leveraging the innate injury-targeting ability of platelets, PT-BsAb effectively guides HSCs to damaged cardiac tissue, thereby facilitating heart repair. In vivo fluorescence imaging revealed that PT-BsAb delivered via inhalation accumulated in the heart at a rate 3.4 times higher than that delivered via intravenous injection, demonstrating significant advantages in cardiac targeting and therapeutic efficacy (Figure 4A).

**Table 2:** Receptors, preparation methods, delivery modes, selected animal models, and application outcomes of OIADD for active targeting of NDDS in various diseases.

Disease	Receptor	Preparation Method and NDDS Type, Size	Mode of Administration	Animal Model	Application Outcomes	Ref.
Lung Cancer	Epidermal growth factor receptor (EGFR)	Modification of gelatin nanoparticles with biotin-labeled epidermal growth factor (EGF), 220 nm	Oral inhalation	Nude mice, in situ lung cancer model constructed with A549 cells	Fluorescence signal analysis showed that the nanoparticles were able to penetrate the tumor; the concentration in the lungs after 1 h and 6 h was 7 and 32 times higher than that of the free drug, respectively.	[126]
	Folate receptor	The solid lipid nanoparticles were formed by encapsulating folic acid and paclitaxel on deacetylated chitosan through PEG grafting, 249 ± 36 nm	Endotracheal	Balb/c mice, in situ lung cancer model constructed with Madison109 cells	The fluorescent signal demonstrated the nanoparticles’ effective tumor infiltration capability. After 1 h and 6 h, the concentration in the lungs was found to be 7 and 32 times higher than that of free drugs, respectively.	[127]
	Transferrin receptor (TfR)	Chemical bonding of adriamycin and PEG to the outer side of a ferritin nanocage (FTn) formed by self-assembly of 24 ferritin heavy chains (FTn/FTn-PEG 2k/DOX), 15.3 ± 3.2	Endotracheal	Balb/c mice, in situ lung cancer model constructed with 3LL cells	The free drug stayed predominantly at the margins of the spheroids, whereas FTn/FTn-PEG 2k/DOX was evenly distributed throughout the tumor spheroids. The median survival of mice treated with free drug was 18 days, while mice treated with FTn/FTn-PEG 2k/DOX had a 60% progression-free survival rate within 60 days.	[128]
Bronchitis	Immunoglobulin Fc fragment receptor	Dexamethasone (Dex) was added to a lipid solution containing the Fc-targeting peptide (FcBP), followed by hydration and the addition of PLGA nanoparticles to form hydrophobic layer-loaded Dex, LNPs with a PLGA core and FcBP modification on the surface (FcBP-NP@Dex), 115–145 nm	Intranasal drip	Balb/c mice, ovalbumin-induced asthma model	Pro-inflammatory cytokine levels (TNF-α, IL-13, IL-4) were significantly lower in the FcBP-NP@Dex group compared to nanoparticles not modified with Fc.	[129]
Cerebral Hemorrhage	Late glycosylation end product receptor	miRNA coupled to cholesterol was loaded onto the surface of RAGE-specific binding peptide (RBP)-modified exosomes by hydrophobic interaction (RBP-Exo/AMO181a-chol), <50 nm	Intranasal drip	SD rats with middle cerebral artery occlusion induced by neck surgery	Infarct volume was quantified by TTC staining; after 24 h, the infarct volume in the unmodified exosome-treated group was approximately 1.6 times that of the RBP-Exo/AMO181a-chol group.	[130]
PD	Acetylcholine receptor (AChE)	Propylene sulfide-polyethylene glycol (PPS-PEG) self-assembled to load curcumin and superparamagnetic iron oxide nanoparticles to form micelles. Octadecyl chains modified with penetrating peptide and acetylcholine receptor protein–rabies virus glycoprotein (RVG29) were embedded in exosomes. The micelles were mixed with exosomes and extruded through an extruder to form exosomes encapsulating micelles, 194.9 nm	Intranasal drip	C57BL/6 mouse, 1-methyl-4-phenyl-1,2,3,6-tetrahydropyridine induced PD model	Within 2–12 h, RVG29-modified exosomes accumulated approximately 1.4 to 2 times higher in the brains of PD mice compared to unmodified exosomes.	[131]
AD	Acetylcholine receptor (AChE)	Co-loading of both siRNAs into MSC-derived exosomes and modification of the exosome surface with a stearylamine-RVG29 peptide conjugate (REXO/BAP@siRNAs NPs), 167.6 nm	Intranasal drip	C57BL/6 mice, triple transgenic AD mouse model	Behavioral observations showed that REXO/BAP@siRNAs NPs were more effective in improving memory in mice compared to exosomes unmodified with peptides.	[132]
Glioma	Transferrin receptor (TfR)	Self-assembly of TfR-targeted T7 peptide coupled to cholesterol into micelles by electrostatic adsorption with siRNA binding in aqueous solution, 56.39 ± 2.44 nm	Intranasal drip	C57BL/6J mice, GL261 cells were used to construct an in situ model of glioma.	Quantitative fluorescence assay for ex vivo imaging of brain tissue in mice 2 h after nasal administration of the micelles showed fluorescence intensity at the tumor site 3.3 times higher than that of free siRNA.	[133]

In addition to stimuli-responsive and ligand–receptor binding targeting strategies, cellular communication capabilities and tissue targeting of drugs can be enhanced through biomimetic design [134,135,136,137,138]. For example, 2′,3′-cyclic guanosine monophosphate–adenosine monophosphate (cGAMP) is a potent activator of the innate immune system, Popowski et al. [139] demonstrated that aerosolizable lung-derived exosomes loaded with cGAMP exhibited superior targeting efficiency towards alveolar macrophages compared to liposomes formulated from lung-mimicking surface-active substances. This enhanced targeting capability results in a more robust immune response by activating the STING pathway. Moreover, the lung distribution of exosomes was approximately 3.7 times higher than that of liposomes, underscoring the potential of exosomes as targeted delivery platforms for vaccines. In addition, bacterial or viral vectors can effectively transport therapeutic molecules to sites of disease through their inherent infection mechanisms or specific tissue affinity [134,136]. For instance, certain biomimetic vectors emulate the structure and invasion mechanisms of SARS-CoV-2 while presenting enhanced safety profiles. Zheng et al. [140] developed a hybrid lipid-based NDDS using DPPC/DPPG/DPPE-COOH/Chol, which closely mimicked the lung surfactant composition to encapsulate poly(I:C), the core genetic material of the virion. This system utilizes a chemical catalytic method that facilitates the connection between the receptor-binding domain (RBD) of SARS-CoV-2 and DPPC-COOH within the lipid-based NDDS, forming a spike protein that closely mimics a virus particle. This biomimetic structural vector was designed to replicate the structure and invasion mechanisms of SARS-CoV-2. Following intranasal administration, the SARS-CoV-2 virus-mimicking vector was shown to effectively induce mucosal immunity as a nanovaccine (Figure 4B).

## 4. The Formulation Process and Aerosolization Device of NDDS

Currently, the aerosolization devices for oral delivery include nebulizers (Neb), pressurized metered-dose inhalers (pMDIs), dry powder inhalers (DPIs), and soft mist inhalers (SMIs); for nasal delivery, aerosolization devices include nasal sprays nasal pMDIs, and nasal DPIs. The aerosols produced by these devices can be classified into gaseous dispersions consisting of solid particles suspended in a gas medium, including inhaled powder and nasal powder formulations, and gaseous dispersions consisting of liquid particles suspended in a gas medium, including inhaled aerosols, inhaled liquid formulations, and corresponding nasal formulations.

Owing to the need for aerosolization in OIADD, NDDS faces unique challenges during the aerosolization process compared to other delivery methods, such as aerosolization stability, deposition, and redisposition. First, the aerosolization stability of NDDS is crucial as it directly affects the uniformity and efficacy of nanomedicine in the aerosol form. Second, the deposition and redisposition issues of NDDS must also be addressed to ensure that nanomedicine can be effectively deposited at the target site and retain its original characteristics after redisposition. The formulation process and aerosolization devices are critical factors that influence the aerosolization stability, deposition, and redisposition of NDDS. For instance, NDDS in liquid systems may experience sedimentation, emulsification, crystal growth (such as Ostwald ripening), particle aggregation, and solid-state transformation, which can significantly impair aerosolization performance [141]. In contrast, transforming a liquid solution into a solid powder can substantially enhance the physical and chemical stability of the NDDS, thereby exhibiting greater stability during the aerosolization process [142]. Furthermore, for an NDDS with stability issues, SMI for solution delivery and DPI for powder delivery may be more promising aerosolization devices [143]. Among the different types of Neb devices, the vibrating mesh nebulizer is generally considered more suitable for NDDS aerosolization than the jet and ultrasonic nebulizers, which may induce structural disruption of the NDDS owing to its low shear force and low heat generation characteristics [144,145]. Therefore, selecting an appropriate formulation process and developing or choosing a compatible aerosolization device is essential for maximizing the therapeutic efficacy of NDDS in OIADD.

### 4.1. Formulation Process

#### 4.1.1. Stabilization

##### Stabilizers

The formulation of an NDDS plays a pivotal role in maintaining structural integrity throughout the aerosolization process. Several studies have explored the relationship between the formulation composition of NDDS and its aerolytic stability. For instance, the factors influencing the stability of liposomes during aerosolization include cholesterol content, PEG concentration, and type and amount of auxiliary lipids. The addition of cholesterol enhances membrane rigidity, thereby providing protection during aerosolization [146]. The inclusion of auxiliary lipids can reduce water migration around the liposomal membrane, which helps to maintain membrane integrity in disordered phases [147]. Moreover, the high PEG density provides a steric barrier that prevents NDDS aggregation, which is vital for maintaining liposomal stability under high shear stress conditions [148]. These findings underscore the importance of optimizing the formulation composition when designing NDDS for aerosolization [149]. By precisely controlling the formulation, the surface properties of NDDS can be optimized, significantly improving their physicochemical attributes, thereby reducing potential drug loss during aerosolization and ensuring efficient drug delivery.

**Figure 5:**
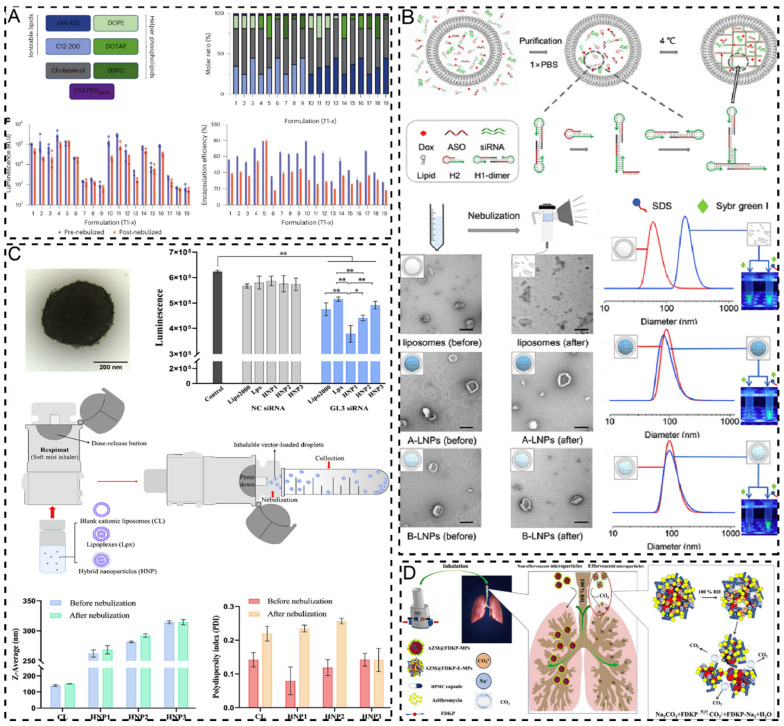
Effect of prescription process on atomization stability, deposition, and redispersion of NDDS. (**A**) Effect of compositional variations of different LNP formulations on mRNA delivery efficiency. By varying the molar ratio of ionizable lipids, auxiliary phospholipids, and cholesterol, 19 formulations of LNPs were prepared, and their encapsulation rates after nebulization and transfection efficiencies in A549 cells were determined. Adapted from ref. [150] with permission. (**B**) Schematic of the preparation of hydrogel-LNPs, stability testing of LNPs during nebulized delivery, demonstrating TEM results before and after nebulization of liposomes, A-LNPs, and B-LNPs, scale bar: 200 nm, as well as DLS results before and after nebulization of liposomes, A-LNPs, and B-LNPs, and permeability changes. Adapted from ref. [160] with permission. (**C**) This figure illustrates the process of nebulizing blank cationic liposomes (CL), Lpx, and HNPs by SMI. The particle size and PDI of these particles were measured both before and after nebulization (*: *p* < 0.05, **: *p* < 0.01). Adapted from ref. [162] with permission. (**D**) Schematic diagram of the composition and redispersion of AZM@FDKP~E-MPs. Adapted from ref. [178] with permission.

In addition to liposomes, studies have reported the impact of formulation composition on the aerosolization stability of LNPs. For example, Jiang et al. [150] observed a decrease in in vitro transfection efficiency and an increase in the particle size of LNPs aerosolized using an Aeroneb vibrating mesh nebulizer, along with increased mRNA encapsulation loss. Cryo-TEM images showed random stacking of lamellar structures on the LNP surface after aerosolization, contrasting sharply with the well-defined spherical shape and electron-dense appearance of LNPs before aerosolization, indicating surface damage during the process. To enhance aerosolization stability, they evaluated LNP formulations based on in vitro transfection efficiency, mRNA encapsulation efficiency, and particle size changes before and after aerosolization. The results revealed that the molar ratio and types of cationic lipids, auxiliary lipids, and cholesterol are critical for forming stable LNPs; using weakly acidic NaAc as a buffer reduced LNP aggregation, and employing bPEG20K as a stabilizer minimized LNP disruption during aerosolization (Figure 5A). Similarly, Zhang et al. [151] found that LNPs containing DSPE exhibited significantly increased aggregation after aerosolization compared to those containing DMG and DMPE.

Specifically, for NDDS using pMDIs as the aerosolization device, stability issues during aerosolization are more pronounced owing to the aggregation induced by the propellant media and mechanical disruption. For instance, during the aerosolization of liposomes, where the propellant HFA is used instead of water as the dispersion medium, the hydrophobic propellant can increase the diffusion rate of phospholipid molecules in a hydrophobic environment, leading to less dense bilayer structures in the liposomes. This makes them more prone to fusion owing to collisions driven by Brownian motion, ultimately forming aggregates [152]. Additionally, the high vapor pressure and mechanical shear forces generated by HFA in pMDIs can disrupt liposome structures and cause drug leakage [152]. Chang et al. [153] developed a reverse micellar system based on lecithin to stabilize protein drugs in pMDIs. Unlike normal micelles formed spontaneously by lecithin in the aqueous phase, this reverse micellar system has a highly polar interior and a low-polarity exterior suitable for encapsulating hydrophilic protein drugs. The formation process of the reverse micellar system involves the spontaneous assembly of lecithin into inner and outer aqueous bilayer structures via hydrophobic interactions, protein–drug adsorption on the surface of the inner and outer aqueous phases, and subsequent cryo-crystallization, which disrupts the hydrophobic interactions of the low-polarity region and forms new lecithin ice crystal phases. Upon solvent removal during freeze-drying, lecithin aligned to form uniformly sized carrier particles with protein drugs loaded in the highly polar region, whereas the low-polarity region was distributed outward. When this reverse micellar system is aerosolized using pMDIs, the outward distribution of the lecithin’s low-polar region enhances compatibility with the low-polar propellant, resolving the stability issues associated with particle aggregation during aerosolization.

##### Core Gelation

The development of hybrid lipid–polymer nanocomposites leverages the beneficial properties of both polymeric and lipid-based NDDS, where the physical crosslinking of polymers within the aqueous core of lipid NDDS can be induced by various triggers, such as light [154], ultrasound [155], pH [156], and ions [157]. Petralito et al. [158] encapsulated polyethylene glycol dimethacrylate (PEG750-DMA or PEG4000-DMA) with different molecular weights in a unilamellar liposome composed of hydrogenated soybean phosphatidylcholine and cholesterol. Upon UV irradiation, free-radical polymerization was initiated, converting the encapsulated material into a hydrogel, thereby enhancing the structural stability of the carrier.

Furthermore, DNA precursors can be crosslinked into hydrogels via continuous hybridization chain reactions, which have also been utilized for constructing hydrogel cores. DNA hydrogels exhibit superior dynamic properties, mechanical strength, and biocompatibility compared to polymeric hydrogels [159]. To enhance the structural stability of the LNPs, Wei et al. [160] developed LNPs doped with a DNA hydrogel (hydrogel-LNPs). These LNPs were prepared by coextruding a mixture of DNA precursors and lipids, followed by ultrafiltration to remove any external DNA. Without the addition of initiators, the DNA within the LNPs gradually matured into a hydrogel over several hours, increasing the strength of the LNPs. The study demonstrated that after aerosolization, the particle size of conventional LNPs increased from 91 to 215 nm, whereas that of hydrogel-LNPs remained virtually unchanged (Figure 5B). Additionally, in leakage detection assays using fluorescently labeled DNA, no significant fluorescence leakage into the solvent was observed for hydrogel-LNPs, indicating excellent aerosolization stability. Thus, hydrogel-LNPs possess robust stability during aerosolization, making them promising candidates for inhalation-based therapeutic delivery.

##### Protective Layer

In the development of aerosolized NDDS, the incorporation of a protective layer, such as a polymeric coating, can significantly enhance the resistance to destabilizing external factors [161]. Wang et al. [162] utilized microfluidic technology to coat cationic liposomes loaded with siRNA using PLGA, producing polymer–lipid hybrid nanoparticles (HNP1, HNP2, HNP3) with progressively thicker PLGA shells (Figure 5C). This study found that HNP3, with the thickest shell, exhibited the most stable particle size and uniformity in dispersion (PDI) following nebulization via a soft mist inhaler (SMI), likely because of the ability of the thicker PLGA shell to counteract dispersion forces during aerosolization. Moreover, storage stability assessments revealed significant aggregation of uncoated nanoparticles within 1 h, whereas HNPs maintained a consistent particle size for over four weeks. Hyaluronic acid (HA) has also been shown to prevent particle aggregation owing to steric hindrance. According to this property, researchers applied ion gel technology to coat chitosan nanoparticles loaded with ferulic acid (FACS) and HA, forming FACHA nanoparticles for asthma treatment. Based on this property, ionic gel technology has been used to coat HA onto the surface of chitosan nanoparticles loaded with ferulic acid to form nanoparticles (FACHA) for asthma treatment [163]. Thermogravimetric analysis after aerosolization via an Aeroneb^®^ Pro vibrating mesh nebulizer indicated that FACHA exhibited superior thermal stability compared to both drug-free chitosan nanoparticles and FACS, suggesting that the HA coating effectively enhanced resistance to nebulizer-induced destabilization.

##### Dry Powderization

It is well established that the development of aerosolized formulations of unstable drugs, such as aerosolized vaccines, by transforming them into solid dry powders can offer improved storage stability. This is particularly advantageous in resource-limited regions that lack cold-chain facilities [164]. Dry powderization also plays a crucial role in stabilizing the NDDS. Ye et al. [165] developed a nanoparticle carrier co-constructed from an adjuvant and structural peptide, with the exterior of the carrier modified with the RBD antigen, yielding R-CNP. R-CNP demonstrated a behavior similar to that of SARS-CoV-2, binding to the host cell receptor ACE2 and inducing specific antibody production in vivo. They then used membrane emulsification to prepare porous PLGA microspheres and encapsulated R-CNP through a simple mixing and mild heating process, ultimately producing a dry powder formulation of R-CNP@M through lyophilization. Stability studies and in vivo experiments revealed that R-CNP@M maintained a consistent aerodynamic diameter and behavior in solution for one month. Furthermore, mice that inhaled R-CNP@M exhibited significantly higher levels of RBD-specific antibodies in the bronchoalveolar lavage fluid (BALF) and nasal wash fluid on day 70 than mice that inhaled R-CNP aerosol. These findings indicate that R-CNP@M dry powder not only possesses excellent aerosolization stability but also holds promise for mucosal immunization applications.

#### 4.1.2. Optimization of Deposition and Redispersion

The deposition requirements for OIADD are tailored according to the target indications because the drug deposition pattern influences drug absorption, retention time, and tissue distribution. USP<1601> includes methods for determining the aerosol lung deposition distribution using the Andersen cascade impactor and next-generation impactor (NGI). The smaller the MMAD within the 1~5 μm range, the higher the probability of the particles reaching the distal branches of the airways, thus resulting in improved lung deposition [166]. Another important evaluation metric is the fine particle fraction (FPF), which generally refers to the percentage of drugs deposited in the lungs. A higher FPF value indicates a higher drug delivery efficiency and greater lung deposition. Although these standards cannot replace in vivo studies, they provide a rationale for further development [167]. Before the immune system clears pathogens, the nasal vestibule, covered with squamous keratinized epithelium and sebaceous glands, serves as a protective barrier. The growing nasal hairs filter out approximately 80% of airborne particles larger than 12.5 μm. About 1.5 cm from the nostrils lies the nasal valve, the narrowest part of the nasal airway. This region has the highest respiratory resistance and airflow velocity, limiting the entry of particles larger than 3 μm through air turbulence and nasal hair vibration [168]. For nasal aerosol devices, the spray pattern and plume geometry of the generated aerosol are FDA-recommended in vitro evaluation parameters for bioavailability and bioequivalence and can serve as critical indicators of drug deposition sites within the nasal cavity [169]. The FDA recommends the use of non-impact methods for determination [170]. With advancements in technologies such as computed tomography, computational fluid dynamics simulations, and 3D printing, more precise and comprehensive solutions for the in vitro evaluation of drug deposition are becoming available. A classic technique for improving the pulmonary deposition of aerosolized drugs is large porous particle (LPP) technology [171]. This technology enables LPPs to have both an appropriate MMAD and the ability to avoid immune clearance in vivo. Common LPP techniques involve encapsulating nanoparticles within PLGA, where the aerodynamic characteristics of the nanoparticles are improved by controlling the type and amount of porogen, thereby increasing the lung deposition rate of the drug [172]. Owing to its highly ordered shape and tunable pore quantity, mesoporous silica offers advantages for controlling aerodynamic properties during the deposition process, although its biocompatibility requires careful evaluation [173]. Additionally, some expandable drug carrier matrices, such as chitosan [174] and gelatin [175], can endow nanoparticles with favorable aerodynamic properties. Nanoparticles prepared using these carrier matrices can remain within an inhalable particle size range in vitro and during transit; however, upon reaching the deep lung and contacting the pulmonary mucus lining, they expand to a larger geometric size. This expansion successfully prevents macrophage clearance and facilitates sustained drug release.

In the powder and suspension forms of NDDS, particle aggregation and agglomeration significantly affected the final deposition efficiency of the drug. Aggregation refers to the formation of stable, irreversible bonds between particles, whereas agglomeration involves weaker, reversible physical forces. Upon aerosol deposition, NDDS typically dissolves in body fluids and releases individual nanoparticles [176,177]. The re-dispersion process of aerosolized NDDS powder generally includes the following steps: (i) NDDS either aggregating or disperse within or on the surface of excipients to form micron-sized aggregates; (ii) fluidization of these aggregates; and (iii) disaggregation into primary nanoparticles. During storage and administration, these micron-sized aggregates act as an intermediate transport system until they reach the lung or nasal mucus lining, where they dissolve and release nanoparticles. Wang et al. [178] developed effervescent microparticles (AZM@FDKP-E-MPs) by dripping an azithromycin (AZM)/sodium carbonate solution into a fumaryl diketopiperazine (FDKP)/citric acid solution, followed by spray drying to treat pneumonia via inhalation. In the trachea, the carboxyl groups on FDKP enable effervescence, breaking the particles into smaller ones and enhancing deep lung deposition and delivery efficiency. To simulate tracheal conditions with high humidity, the aerosolized powder was directed into the ((NGI) at 100% relative humidity. By varying the ratio of carboxyl groups in FDKP and the molar ratio of citric acid to sodium carbonate, the optimal formulation displayed better flowability, smaller aerodynamic diameter (D_ae_ of 6.376 ± 0.825 μm), and a higher fine particle fraction (FPF of 67.52 ± 0.12%). The in vivo results in mice demonstrated that AZM@FDKP-E-MPs achieved significantly higher deep lung deposition than non-effervescent formulations within the first four hours post-administration. Malamatari et al. [152], in a comprehensive review, discussed various preparation techniques for nanoparticle-based dry powders, including traditional spray drying, freeze drying, spray freeze drying, nanospray drying, and supercritical fluid-assisted spray drying. Each technique employs different matrices to satisfy the specific processing requirements. For example, polyols [179,180], polysaccharides [181], and amino acids [182] used as matrices during traditional spray drying effectively prevent nanoparticle aggregation due to heat damage. Freeze drying requires cryoprotectants to stabilize the nanoparticles by forming hydrogen bonds, thus preventing irreversible aggregation due to phase separation [183]. In a study by Fukushige et al. [184], siRNA was complexed with protamine and DNA to form a dense core with a negative charge and then coated with a cationic liposome-derived lipid bilayer to create a liposome–protamine–DNA complex (LPD). Coating this complex with hyaluronic acid (HA) and freeze-drying produced a powder (LPDH). Research has shown that the particle size of the LPDH powder after re-dispersion was unchanged from its original form, while the LPD powder size increased by 2.12 times. This indicates that the freeze-drying process enhanced the re-dispersion properties of the LPDH powder, likely owing to HA’s cryoprotective role of HA during the freeze-drying process, aiding in aerosol re-dispersion post-inhalation.

### 4.2. Aerosolization Devices

In 1778, British physician John Mudge first coined the term “Inhaler” and designed the “Mudge inhaler”. He advised that this inhaler should be used exclusively with the medication mentioned in his article because combining it with other drugs might diminish its efficacy [185]. This foresight anticipated the importance of the “drug-device combination” concept in OIADD. Over the past six to seven decades, aerosolized devices have undergone significant evolution and innovation characterized by miniaturization, enhanced performance, and improved user-friendliness. For example, nebs have become portable, DPIs have integrated auxiliary aerosolization power to reduce user reliance on inhalation techniques, and pMDIs have transitioned from harmful CFCs to environmentally friendly HFA propellants. Nasal aerosolization devices feature dosing mechanisms that ensure precision. Furthermore, sensor-based inhaler-assistive devices have been developed to monitor the peak inspiratory flow and inhalation volume, track medication intake, and record usage time and frequency. These advancements have enhanced treatment safety and efficacy by providing real-time feedback and personalized guidance to both patients and healthcare providers [186].

Nonetheless, the fact that existing aerosolization devices are mainly based on aerosolizing conventional solutions or suspensions rather than being specifically designed for NDDS has inspired researchers to develop novel aerosolization devices. For example, as the only approved aerosolized nanomedicine delivery system on the market today, amikacin liposomal inhalation suspension Arikayce^®^ (Insmed Incorporated) replaced the original eFlow^®^ nebulizer in its Phase III clinical trial with a specially customized Lamira^®^ nebulizer. The Lamira^®^ nebulizer uses a customized drug reservoir (holds a dose of 8.4 mL), a custom aerosol head specifically designed for Arikayce^®^ nebulization, and a valved aerosol chamber, which allows the liposomes to be nebulized to produce an aerosol of uniform size with an MMAD of 4.7 μm, which facilitates deposition in the lungs [187]. Currently, aerosolization devices for NDDS are evolving towards gentler and more efficient designs. By optimizing the aerosolization mechanisms and device architecture, these advancements aim to maximize the preservation of drug activity and NDDS integrity, thereby enhancing therapeutic outcomes.

#### 4.2.1. Stability

**Figure 6:**
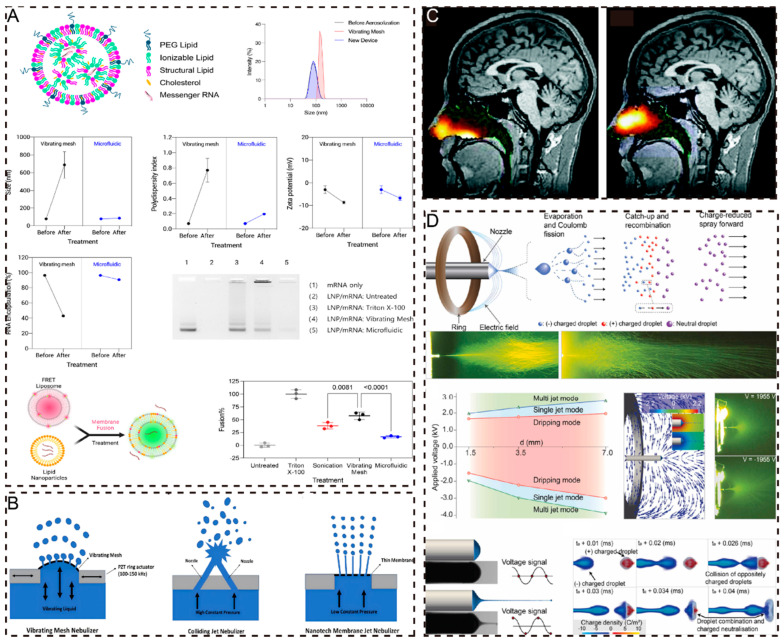
Aerosolization devices to improve NDDS stability, deposition, and redispersion. (**A**) Schematic representation of individual LNP/mRNAs including PEG lipids (blue), ionizable lipids (green), structured lipids (pink), and cholesterol (yellow), demonstrating representative size distributions of LNP/mRNAs under different treatments: untreated (grey), vibrating mesh (red), and MAP (blue), as well as the particle sizes of the LNPs/mRNAs after aerosolization, the changes in zeta potentials, changes in encapsulation rate, and schematic diagrams and results of FRET-based lipid membrane fusion assay are shown. Adapted from ref. [188] with permission. (**B**) Principle of NM atomization. Adapted from ref. [189] with permission. (**C**) Demonstration of intranasal deposition after 2 min of administration using a conventional liquid spray pump and Opt-Powder by gamma camera image information. Adapted from ref. [197] with permission. (**D**) demonstrated the concept of in-flight electrically neutralized electrospray, including its working principle, experimental images, simulation of spray morphology in DC and AC modes in comparison with experiment, and simulated time series of the neutralization process. Adapted from ref. [198] with permission.

Kim et al. [188] developed a microfluidic aerosolization platform (MAP). This device features an array of 960 droplet injectors, each individually controllable and activated by short electrical pulses to the heater elements, generating microbubbles that precisely eject droplets containing LNPs/mRNAs at low operational frequencies. This innovative design minimizes the exposure of nanoparticles to destructive shear forces, thereby enhancing the stability of the NDDS during aerosolization (Figure 6A). Cryo-transmission electron microscopy revealed that MAP preserved the morphology of the LNPs more effectively than the deformation and dissociation observed with vibrating-mesh nebs. In vitro transfection experiments demonstrated that MAP achieved transfection efficiencies that were approximately 5~187 times higher than those obtained with vibrating-mesh Nebs. Additionally, a recently published study introduced a novel nebulization method termed nanotechnology membrane (NM) for effective SARS-CoV-2 mRNA vaccine delivery [189]. This method also operates at lower energy levels to minimize structural damage to the NDDS. The principle can be succinctly described as follows: Under low pressure (5~20 bar), a liquid passes through a film with approximately 50~100 uniformly sized nanopores, producing equal-sized jets that disintegrate into droplets and form aerosols [189]. Researchers compared NM with two commonly used vibrating mesh Nebs (Pari eflow^®^ and Aerogen Solo^®^) and the only commercially available SMI (Respimat^®^). They found that NM nebulized the LNPs with minimal size alteration, leading to a more robust immune response upon administration (Figure 6B).

Traditional ultrasonic nebulizers, which typically operate at 100 kHz and require more than 10 watts of power, can inflict shear and cavitation damage to unstable drugs suspended in liquids [190]. Surface acoustic wave (SAW) Neb is a modified ultrasonic device that utilizes an acoustic wave with a nanometer-range displacement amplitude that propagates along the surface of a single-crystal piezoelectric substrate. This wave decays rapidly with depth, thereby limiting the energy transfer and reducing its impact on the liquid. Cortez-Jugo et al. [191] demonstrated that SAW-aerosolized nanoparticles formed from siRNA-PEI or cationic lipid nanocomplexes maintained their original particle size and zeta potential, thereby highlighting the potential of SAWs in producing stable aerosolized NDDS.

In SMI, aerosol generation is achieved through the impact of two jets resulting from the compression of a uniblock micrometer chip by a spring mechanism. Klein et al. [192] reported that SMIs caused significantly less damage to liposomes than two commercially available vibrating mesh nebs. Similarly, Wang et al. [162] demonstrated the superior performance of SMI for nebulizing polymer–lipid hybrid nanoparticles (HNPs).

#### 4.2.2. Optimization of Deposition and Redispersion

In intranasal drug delivery, the efficiency of drug deposition is highly dependent on parameters such as plume angle and velocity because of the direct or adjacent pathways of administration to the lesion. Selecting appropriate parameters is critical for maximizing the therapeutic efficacy [193]. Le Guellec et al. [194] provided a comprehensive discussion of intranasal drug deposition. To ensure that drugs effectively reach target areas such as the olfactory and trigeminal regions, specialized aerosolizing devices that control droplet size distribution, plume angle, and spray pattern are essential for navigating complex nasal structures. For example, Pina Costa et al. [195] designed a specialized nasal spray to deliver an in situ gel solution loaded with diazepam (DZP-NLC-Pec) to treat epilepsy. Deposition studies using 3D-printed models of the human nasal cavity revealed that DZP-NLC-Pec deposition was influenced by the aerosoliairflow and angle parameters of the aerolyzing device, with approximately 47% of the optimized DZP-NLC-Pec successfully deposited in the olfactory region. Maaz et al. [196] explored the penetration characteristics of pMDIs in nose-to-brain drug delivery and showed that the device enabled nanoparticles to penetrate narrow nasal valves and achieve effective delivery to the olfactory region. Compared to propellant-free aerosolized NP solutions, the pMDIs group achieved over 80% drug penetration across the nasal epithelial cells, compared to less than 60% for the propellant-free group, indicating a superior deposition effect with pMDIs. Djupesland et al. [197] constructed regional nasal deposition achieved by a novel bidirectional nasal drug delivery device, Opt-Powder, with that of a conventional liquid spray pump via dynamic gamma camera imaging. The Opt-Powder device leverages the mechanism of soft palate closure during exhalation to achieve efficient drug deposition in the upper posterior nasal cavity. The results showed almost no lung deposition with opt-powder, with drug deposition rates in the upper posterior nasal cavity being approximately nine times higher than that with conventional nasal sprays and three times higher in the middle upper nasal cavity region (Figure 6C).

For certain NDDS, electrostatic build-up during aerosolization should not be overlooked, as it can alter the surface properties and functionality of the NDDS. Vu et al. [198] developed an electrically neutralized Neb that generated sprays of oppositely charged droplets by applying an alternating current. As the charged droplets decelerate, a reverse electric field accelerates the oppositely charged droplets, causing them to mix and neutralize the charges (Figure 6D). The neutralized droplets then continue to move owing to momentum, which is unaffected by the electric field.

The traditional DPI consists of two tangential inlets of opposite diameters, with the drug powder contained in a hemispherical dosing cup topped by the patient’s mouth for inhalation. When inhaled, air enters the DPI through the tangential inlets, generating a rotating axial airflow that fluidizes the powder in the dosing cup and carries the drug agglomerates into the airflow. Chaugule et al. [199] designed three “counter-vortex” DPIs, incorporating six tangential inlets at the bottom of the dosing cup to generate a counterclockwise primary rotational suction airflow and another six inlets in the opposite direction above, generating a clockwise secondary counter-rotational suction airflow. This design effectively reduced drug aggregation during aerosolization. In vitro deposition studies demonstrated that the “anti-vortex” DPI significantly reduced drug deposition losses in the NGI’s induction port compared to conventional DPIs while increasing drug deposition during the initial phase of the NGI. Although the drugs employed do not incorporate nanotechnology, they still offer valuable insights into aerosolized NDDS.

To address the agglomeration–redispersion nature of powder aerosols, optimizing DPI design can be beneficial. Lee et al. [200] incorporated mouthpieces with helical channels into DPI to induce strong vortexing and facilitate drug redispersion on the carrier surface. Evaluations using an Anderson cascade impactor for aflumoterol aerosols and budesonide aerosols showed that the non-spiral device produced MMADs of 6.7 ± 0.1 μm and 6.9 ± 0.1 μm with FPFs of 39.9 ± 3.4% and 44.7 ± 2.0%, respectively. In contrast, the spiral device significantly improved aerosol particle size distribution and deposition, with MMADs of 2.6 ± 0.5 μm and 3.3 ± 0.3 μm, and FPFs of 64.1 ± 1.1% and 64.4 ± 1.0%, respectively.

## 5. Summary and Prospect

**Figure 7:**
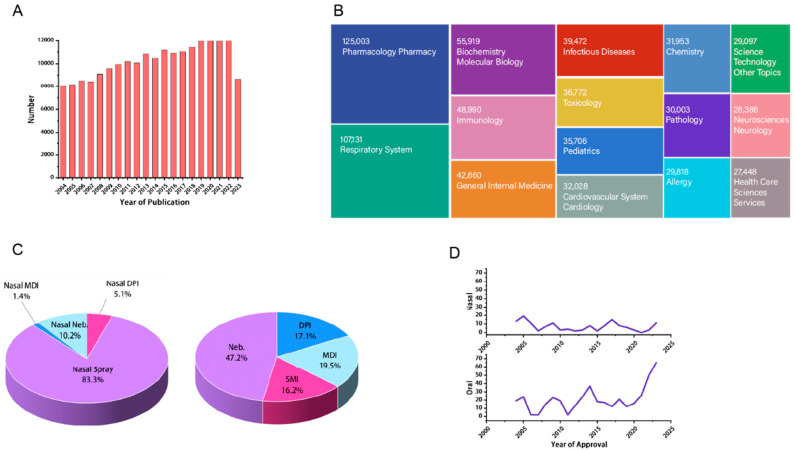
Statistical trends in the development of OIADD. (**A**) Number of OIADD-related publications from 2004 to 2023. (**B**) Key focus areas in OIADD research. (**C**) Proportion of clinical formulation studies. (**D**) Number of OIADD-related drugs approved by the FDA from 2004 to 2023.

A comprehensive statistical scrutiny of advancements in the field of OIADD from 2004 to 2023 was conducted via the Web of Science database. This analysis revealed a consistent increase in the number of OIADD-related publications (Figure 7A), indicating a significant increase in research interest in this domain. Respiratory, infectious, cardiovascular, and among the popular areas of OIADD research (Figure 7B), respiratory, infectious, cardiovascular, and neurological diseases occupy a prominent position, highlighting the urgent need for inhaled therapies in these areas. Statistical data from the PubMed database (Figure 7C) demonstrated the distribution of clinical studies on different OIADD formulations, with nasal sprays dominating nasal aerosolized drugs (83.3%) and Neb accounting for the majority of oral inhalation drugs (47.2%). This finding suggests that despite growing research on novel drug delivery systems and drugs, clinical practice still relies heavily on traditional and well-established formulations. Furthermore, using the Drugs@FDA.gov database, the authors examined the number of FDA-approved OIADD by searching for “Inhalation” and “Nasal” as routes of administration (Figure 7D). The results indicated a general upward trend in the approval of oral inhalation drugs, whereas the number of approved nasal aerosolized drugs remained relatively low and did not exhibit the significant growth pattern observed for oral inhalation drugs.

Despite the challenges plaguing OIADD, NDDS have significantly enhanced drug delivery efficiency and precision through various mechanisms, reducing side effects and improving patient compliance. The integration of NDDS with advanced aerosolization devices offers promising prospects for treating a wide range of diseases.

In summary, although the research and application of OIADD have expanded in both scope and depth, its clinical translation and implementation still face notable limitations. Possible reasons include: (i) The relatively long development cycle of OIADD, as the transition from laboratory research to clinical application requires complex approval processes; (ii) The acceptance of OIADD by physicians and patients remains relatively low; (iii) The cost-effectiveness of OIADD may not yet meet the level required for widespread market adoption; (iv) Strict regulatory and policy scrutiny further delays its clinical application; (v) The application of nanomedicine in OIADD still faces significant challenges, such as the development of in vitro models and the establishment of in vivo evaluation standards.

In recent years, OIADD has transcended its traditional role in respiratory disease drug delivery, playing a crucial role in delivering therapeutic agents for brain diseases, cardiovascular diseases, and other conditions. With advancements in various aspects of nanomedicine, OIADD is expected to play an increasingly important role in modern healthcare, driving innovation and progress in therapeutic strategies.

## Figures and Tables

**Table 1:** Summary of indications, trade names, active ingredients, dosage forms, companies, and year of approval for FDA-approved OIADD products for the treatment of other diseases.

Indications	Brand	API	Dosage Form	Company	Market Launch
Endometriosis	Synarel^®^	Nafarelin	Nasal Spray	Pfizer	1990
Pain Relief	Sprix^®^	Ketorolac Tromethamine	Nasal Spray	Roxro	2010
Type II Diabetes	Afrezza^®^	Insulin	Inhalation Powder	Mannkind	2014
Opioid Overdose	Narcan^®^	Naloxone	Nasal Spray	Adapt Pharma	2015
Dental Anesthesia	Kovanaze^®^	Tetracaine/Oxymetazoline	Nasal Spray	St. Renatus	2016
Hypoglycemia	Baqsimi^®^	Glucagon	Nasal Powder	Amphastar	2019
Diabetic Gastroparesis	Gimoti^®^	Metoclopramide	Nasal Spray	Evoke Pharma	2020
Xerophthalmia	Tyrvaya^®^	Varenicline	Nasal Spray	Oyster Point Pharma	2021
Radiographic Examination	Xenoview^®^	Xenon Xe 129 Hyperpolarized	Inhalation Gas	Polarean	2022

## References

[1] Sanders M. (2007). Inhalation therapy: An historical review. Prim. Care Respir. J..

[2] Marx D., Williams G., Birkhoff M., Vallisuta O., Olimat S. (2015). Intranasal Drug Administration—An Attractive Delivery Route for Some Drugs. Drug Discovery and Development From Molecules to Medicine.

[3] Aggarwal R., Cardozo A., Homer J.J. (2004). The assessment of topical nasal drug distribution. Clin. Otolaryngol. Allied Sci..

[4] Foo M.Y., Cheng Y.S., Su W.C., Donovan M.D. (2007). The Influence of Spray Properties on Intranasal Deposition. J. Aerosol Med..

[5] Kayarkar R., Clifton N.J., Woolford T.J. (2002). An evaluation of the best head position for instillation of steroid nose drops. Clin. Otolaryngol. Allied Sci..

[6] Vasudev M., Torabi S.J., Michelle L., Meller L.L.T., Birkenbeuel J.L., Roman K.M., Nguyen T.V., Kuan E.C. (2023). The rising cost of rhinologic medications. Ann. Allergy Asthma Immunol..

[7] (2023). Global Initiative for Chronic Obstructive Lung Disease, Global Strategy for the Diagnosis, Management and Prevention of Chronic Obstructive Pulmonary Disease. https://goldcopd.org/2023-gold-report-2.

[8] (2023). Global Initiative for Asthma, Global Strategy for Asthma Management and Prevention. https://ginasthma.org/.

[9] Ferguson G.T., Rabe K.F., Martinez F.J., Fabbri L.M., Wang C., Ichinose M., Bourne E., Ballal S., Darken P., DeAngelis K. (2018). Triple therapy with budesonide/glycopyrrolate/formoterol fumarate with co-suspension delivery technology versus dual therapies in chronic obstructive pulmonary disease (KRONOS): A double-blind, parallel-group, multicentre, phase 3 randomised controlled trial. Lancet Respir. Med..

[10] Milara J., Navarro A., Almudever P., Lluch J., Morcillo E., Cortijo J. (2011). Oxidative stress-induced glucocorticoid resistance is prevented by dual PDE3/PDE4 inhibition in human alveolar macrophages. Clin. Exp. Allergy.

[11] Kuhn R.J. (2001). Formulation of Aerosolized Therapeutics. Chest.

[12] Vazquez-Espinosa E., Marcos C., Alonso T., Giron R., Gomez-Punter R., Garcia-Castillo E., Zamora E., Cisneros C., Garcia J., Valenzuela C. (2016). Tobramycin inhalation powder (TOBI Podhaler^®®^) for the treatment of lung infection in patients with cystic fibrosis. Expert Rev. Anti-Infect. Ther..

[13] Parkins M.D., Elborn J.S. (2010). Aztreonam lysine: A novel inhalational antibiotic for cystic fibrosis. Expert Rev. Respir. Med..

[14] Sandifer B.L., Brigham K.L., Lawrence E.C., Mottola D., Cuppels C., Parker R.E. (2005). Potent effects of aerosol compared with intravenous treprostinil on the pulmonary circulation. J. Appl. Physiol..

[15] Horie S., McNicholas B., Rezoagli E., Pham T., Curley G., McAuley D., O’Kane C., Nichol A., Santos C.D., Rocco P.R.M. (2020). Emerging pharmacological therapies for ARDS: COVID-19 and beyond. Intensive Care Med..

[16] Battaglini D., Fazzini B., Silva P.L., Cruz F.F., Ball L., Robba C., Rocco P.R.M., Pelosi P. (2023). Challenges in ARDS Definition, Management, and Identification of Effective Personalized Therapies. J. Clin. Med..

[17] Beitler J.R., Thompson B.T., Baron R.M., Bastarache J.A., Denlinger L.C., Esserman L., Gong M.N., Lavange L.M., Lewis R.J., Marshall J.C. (2022). Advancing precision medicine for acute respiratory distress syndrome. Lancet Respir. Med..

[18] Bos L.D.J., Ware L.B. (2022). Acute respiratory distress syndrome: Causes, pathophysiology, and phenotypes. Lancet.

[19] Wu T., Zuo Z.H., Kang S.T., Jiang L.P., Luo X., Xia Z.X., Liu J., Xiao X.J., Ye M., Deng M.C. (2020). Multi-organ Dysfunction in Patients with COVID-19: A Systematic Review and Meta-analysis. Aging Dis..

[20] Liu C., Ma Y., Su Z.L., Zhao R.Z., Zhao X.L., Nie H.G., Xu P., Zhu L.L., Zhang M., Li X.M. (2018). Meta-analysis of preclinical studies of fibrinolytic therapy for acute lung injury. Front. Immunol..

[21] Bando M., Homma S., Date H., Kishi K., Yamauchi H., Sakamoto S., Miyamoto A., Goto Y., Nakayama T., Azuma A. (2024). Japanese guidelines for the treatment of idiopathic pulmonary fibrosis 2023: Revised edition. Respir. Investig..

[22] Dheda K., Lange C. (2022). A revolution in the management of multidrug-resistant tuberculosis. Lancet.

[23] Hirsch F.R., Scagliotti G.V., Mulshine J.L., Kwon R., Curran W.J., Wu Y.L., Paz-Ares L. (2017). Lung cancer: Current therapies and new targeted treatments. Lancet.

[24] Wang M., Herbst R.S., Boshoff C. (2021). Toward personalized treatment approaches for non-small-cell lung cancer. Nat. Med..

[25] Nguyen L., Hindiyeh N., Ray S., Vann R.E., Aurora S.K. (2023). The Gut-brain Connection and Episodic Migraine: An Update. Curr. Pain Headache Rep..

[26] Aurora S.K., Shrewsbury S.B., Ray S., Hindiyeh N., Nguyen L. (2021). A link between gastrointestinal disorders and migraine: Insights into the gut–brain connection. J. Headache Pain.

[27] Lipp M.M., Hickey A.J., Langer R., LeWitt P.A. (2021). A technology evaluation of CVT-301 (Inbrija): An inhalable therapy for treatment of Parkinson’s disease. Expert Opin. Drug Deliv..

[28] Paul M., Lau R. (2020). Potentials and challenges of Levodopa particle formulation for treatment of Parkinson’s disease through intranasal and pulmonary delivery. Adv. Powder Technol..

[29] Schaefer B.B.M.L., Silver W.L., Finger T.E. (2002). Trigeminal collaterals in the nasal epithelium and olfactory bulb: A potential route for direct modulation of olfactory information by trigeminal stimuli. J. Comp. Neurol..

[30] Thorne R.G., Pronk G.J., Padmanabhan V., Frey W.H. (2004). Delivery of insulin-like growth factor-I to the rat brain and spinal cord along olfactory and trigeminal pathways following intranasal administration. Neuroscience.

[31] Lochhead J.J., Wolak D.J., Pizzo M.E., Thorne R.G. (2015). Rapid Transport within Cerebral Perivascular Spaces Underlies Widespread Tracer Distribution in the Brain after Intranasal Administration. J. Cereb. Blood Flow Metab..

[32] Lochhead J.J., Thorne R.G. (2012). Intranasal delivery of biologics to the central nervous system. Adv. Drug Deliv. Rev..

[33] Safirstein B.E., Ellenbogen A., Zhao P., Henney H.R., Kegler-Ebo D.M., Oh C. (2020). Pharmacokinetics of inhaled levodopa administered with oral carbidopa in the fed state in patients with Parkinson’s disease. Clin. Ther..

[34] Yang C.P., Liang C.S., Chang C.M., Yang C.C., Shih P.H., Yau Y.C., Tang K.T., Wang S.J. (2021). Comparison of New Pharmacologic Agents With Triptans for Treatment of Migraine: A Systematic Review and Meta-analysis. JAMA Netw. Open.

[35] Munjal S., Brand-Schieber E., Allenby K., Spierings E.L.H., Cady R.K., Rapoport A.M. (2017). A multicenter, open-label, long-term safety and tolerability study of DFN-02, an intranasal spray of sumatriptan 10 mg plus permeation enhancer DDM, for the acute treatment of episodic migraine. J. Headache Pain.

[36] Munjal S., Gautam A., Offman E., Brand-Schieber E., Allenby K., Fisher D.M. (2016). A Randomized Trial Comparing the Pharmacokinetics, Safety, and Tolerability of DFN-02, an Intranasal Sumatriptan Spray Containing a Permeation Enhancer, With Intranasal and Subcutaneous Sumatriptan in Healthy Adults. Headache.

[37] Lipton R.B., Croop R., Stock D.A., Madonia J., Forshaw M., Lovegren M., Mosher L., Coric V., Goadsby P.J. (2023). Safety, tolerability, and efficacy of zavegepant 10 mg nasal spray for the acute treatment of migraine in the USA: A phase 3, double-blind, randomised, placebo-controlled multicentre trial. Lancet Neurol..

[38] Larik M.O., Iftekhar M.A., Syed B.U., Ansari O., Ansari M. (2023). Nasal spray (Zavegepant) for migraines: A mini-review. Ann. Med. Surg..

[39] Bouw M.R., Chung S.S., Gidal B., King A., Tomasovic J., Wheless J.W., Ess P.J.V. (2021). Clinical pharmacokinetic and pharmacodynamic profile of midazolam nasal spray. Epilepsy Res..

[40] Henney H.R., Sperling M.R., Rabinowicz A.L., Bream G., Carrazana E.J. (2014). Assessment of pharmacokinetics and tolerability of intranasal diazepam relative to rectal gel in healthy adults. Epilepsy Res..

[41] Wei Y., Feng Y., Yazdi M.D., Yin K., Castro E., Shtein A., Qiu X., Peralta A.A., Coull B.A., Dominici F. (2024). Exposure-response associations between chronic exposure to fine particulate matter and risks of hospital admission for major cardiovascular diseases: Population based cohort study. BMJ.

[42] Rajagopalan S., Al-Kindi S.G., Brook R.D. (2018). Air pollution and cardiovascular disease: JACC state-of-the-art review. J. Am. Coll. Cardiol..

[43] Li C.C., Naveed M., Dar K., Liu Z.W., Baig M.M.F.A., Lv R.D., Saeed M., Chen D.D., Yu F., Zhou X.H. (2021). Therapeutic advances in cardiac targeted drug delivery: From theory to practice. J. Drug Target..

[44] Miragoli M., Ceriotti P., Iafisco M., Vacchiano M., Salvarani N., Alogna A., Carullo P., Ramirez-Rodríguez G.B., Patrício T., Esposti L.D. (2018). Inhalation of peptide-loaded nanoparticles improves heart failure. Sci. Transl. Med..

[45] Liu C., Chen L.Y., Ma Y.C., Hu K.Y., Wu P., Pan L.N., Chen H.Y., Li L.L., Hu H.Y., Zhang J.X. (2021). Pulmonary circulation-mediated heart targeting for the prevention of heart failure by inhalation of intrinsically bioactive nanoparticles. Theranostics.

[46] Kreyling W.G., Semmler-Behnke M., Takenaka S., Mo W. (2013). Differences in the biokinetics of inhaled nano-versus micrometer-sized particles. Acc. Chem. Res..

[47] Gizurarson S. (2015). The Effect of Cilia and the Mucociliary Clearance on Successful Drug Delivery. Biol. Pharm. Bull..

[48] Cohen N.A. (2006). Sinonasal Mucociliary Clearance in Health and Disease. Ann. Otol. Rhinol. Laryngol..

[49] Sahin-Yilmaz A., Naclerio R.M. (2011). Anatomy and Physiology of the Upper Airway. Proc. Am. Thorac. Soc..

[50] Chater P.I., Wilcox M.D., Pearson J.P. (2018). Efficacy and safety concerns over the use of mucus modulating agents for drug delivery using nanoscale systems. Adv. Drug Deliv. Rev..

[51] Mastorakos P., Da Silva A.L., Chisholm J., Song E., Choi W.K., Boyle M.P., Morales M.M., Hanes J., Suk J.S. (2015). Highly compacted biodegradable DNA nanoparticles capable of overcoming the mucus barrier for inhaled lung gene therapy. Proc. Natl. Acad. Sci. USA.

[52] Bansil R., Turner B.S. (2006). Mucin structure, aggregation, physiological functions and biomedical applications. Curr. Opin. Colloid Interface Sci..

[53] Mansfield E.D., de la Rosa V.R., Kowalczyk R.M., Grillo I., Hoogenboom R., Sillence K., Hole P., Williams A.C., Khutoryanskiy V.V. (2016). Side chain variations radically alter the diffusion of poly(2-alkyl-2-oxazoline) functionalised nanoparticles through a mucosal barrier. Biomater. Sci..

[54] Ma Y.B., Guo Y.Y., Liu S., Hu Y., Yang C., Cheng G., Xue C.Y., Zuo Y.Y., Sun B.B. (2023). pH-Mediated mucus penetration of zwitterionic polydopamine-modified silica nanoparticles. Nano Lett..

[55] Yang M., Lai S.K., Yu T., Wang Y.Y., Happe C., Zhong W., Zhang M., Anonuevo A., Fridley C., Hung A. (2014). Nanoparticle penetration of human cervicovaginal mucus: The effect of polyvinyl alcohol. J. Control. Release.

[56] Yang M., Lai S.K., Wang Y.Y., Zhong W., Happe C., Zhang M., Fu J., Hanes J. (2011). Biodegradable Nanoparticles Composed Entirely of Safe Materials that Rapidly Penetrate Human Mucus. Angew. Chem. Int. Ed..

[57] Luo T., Loira-Pastoriza C., Patil H.P., Ucakar B., Muccioli G.G., Bosquillon C., Vanbever R. (2016). PEGylation of paclitaxel largely improves its safety and anti-tumor efficacy following pulmonary delivery in a mouse model of lung carcinoma. J. Control. Release.

[58] Mitchell M.J., Billingsley M.M., Haley R.M., Wechsler M.E., Peppas N.A., Langer R. (2021). Engineering precision nanoparticles for drug delivery. Nat. Rev. Drug Discov..

[59] Wu J., Zhai T.S., Sun J., Yu Q.S., Feng Y.C., Li R.W., Wang H., Ouyang Q.H., Yang T.T., Zhan Q.Y. (2022). Mucus-permeable polymyxin B-hyaluronic acid/poly(lactic-co-glycolic acid) nanoparticle platform for the nebulized treatment of lung infections. J. Colloid Interface Sci..

[60] Li D.J., Zhao A., Zhu J.F., Wang C.J., Shen J.J., Zheng Z.X., Pan F., Liu Z., Chen Q., Yang Y. (2023). Inhaled Lipid Nanoparticles Alleviate Established Pulmonary Fibrosis. Small.

[61] Laffleur F., Hintzen F., Shahnaz G., Rahmat D., Leithner K., Bernkop-Schnürch A. (2014). Development and in vitro evaluation of slippery nanoparticles for enhanced diffusion through native mucus. Nanomedicine.

[62] Schuster B.S., Suk J.S., Woodworth G.F., Hanes J. (2013). Nanoparticle diffusion in respiratory mucus from humans without lung disease. Biomaterials.

[63] Bao C., Liu B., Li B., Chai J.J., Zhang L.W., Jiao L.L., Li D., Yu Z.Q., Ren F.Z., Shi X.H. (2020). Enhanced Transport of Shape and Rigidity-Tuned α-Lactalbumin Nanotubes across Intestinal Mucus and Cellular Barriers. Nano Lett..

[64] Fan Q., Li Z.H., Yin J., Xie M., Cui M.R., Fan C.H., Wang L.H., Chao J. (2023). Inhalable pH-responsive DNA tetrahedron nanoplatform for boosting anti-tumor immune responses against metastatic lung cancer. Biomaterials.

[65] Garcia-Mouton C., Hidalgo A., Cruz A., Pérez-Gil J. (2019). The Lord of the Lungs: The essential role of pulmonary surfactant upon inhalation of nanoparticles. Eur. J. Pharm. Biopharm..

[66] Leo V.D., Ruscigno S., Trapani A., Gioia S.D., Milano F., Mandracchia D., Comparelli R., Castellani S., Agostiano A., Trapani G. (2018). Preparation of drug-loaded small unilamellar liposomes and evaluation of their potential for the treatment of chronic respiratory diseases. Int. J. Pharm..

[67] Nafee N., Husari A., Maurer C.K., Lu C., Rossi C., Steinbach A., Hartmann R.W., Lehr C.M., Schneider M. (2014). Antibiotic-free nanotherapeutics: Ultra-small, mucus-penetrating solid lipid nanoparticles enhance the pulmonary delivery and anti-virulence efficacy of novel quorum sensing inhibitors. J. Control. Release.

[68] Wang B.H., Gao Y.W., Sun L.L., Xue M., Wang M.J., Zhang Z.Z., Zhang L.R., Zhang H.L. (2023). Inhaled pulmonary surfactant biomimetic liposomes for reversing idiopathic pulmonary fibrosis through synergistic therapeutic strategy. Biomaterials.

[69] Hoy S.M. (2021). Amikacin liposome inhalation suspension in refractory *Mycobacterium avium* complex lung disease: A profile of its use. Clin. Drug Investig..

[70] Martini L.B., Sulmona C., Brambilla L., Rossi D. (2023). Cell-Penetrating Peptides as Valuable Tools for Nose-to-Brain Delivery of Biological Drugs. Cells.

[71] Khalil I.A., Kimura S., Sato Y., Harashima H. (2018). Synergism between a cell penetrating peptide and a pH-sensitive cationic lipid in efficient gene delivery based on double-coated nanoparticles. J. Control. Release.

[72] Yan X.X., Lin L., Li S.R., Wang W.L., Chen B.G., Jiang S.N., Liu S.R., Ma X.J., Yu X.F. (2020). Arginine-rich peptide based nanoparticles with bridge-like structure: Enhanced cell penetration and tumor therapy effect. Chem. Eng. J..

[73] Torchilin V.P., Levchenko T.S., Rammohan R., Volodina N., Papahadjopoulos-Sternberg B., D’Souza G.G.M. (2003). Cell transfection in vitro and in vivo with nontoxic TAT peptide-liposome–DNA complexes. Proc. Natl. Acad. Sci. USA.

[74] Kleemann E., Neu M., Jekel N., Fink L., Schmehl T., Gessler T., Seeger W., Kissel T. (2005). Nano-carriers for DNA delivery to the lung based upon a TAT-derived peptide covalently coupled to PEG-PEI. J. Control. Release.

[75] Mahri S., Hardy E., Wilms T., De Keersmaecker H., Braeckmans K., De Smedt S., Bosquillon C., Vanbever R. (2021). PEGylation of recombinant human deoxyribonuclease I decreases its transport across lung epithelial cells and uptake by macrophages. Int. J. Pharm..

[76] Osman G., Rodriguez J., Chan S.Y., Chisholm J., Duncan G., Kim N., Tatler A.L., Shakesheff K.M., Hanes J., Suk J.S. (2018). PEGylated enhanced cell penetrating peptide nanoparticles for lung gene therapy. J. Control. Release.

[77] Leal J., Peng X.J., Liu X.Q., Arasappan D., Wylie D.C., Schwartz S.H., Fullmer J.J., McWilliams B.C., Smyth H.D.C., Ghosh D. (2020). Peptides as surface coatings of nanoparticles that penetrate human cystic fibrosis sputum and uniformly distribute in vivo following pulmonary delivery. J. Control. Release.

[78] Yang M.Y., Han M.M., Tang L., Bi Y.Y., Li X.N., Jeong J.H., Wang Y., Jiang H.L. (2024). Dual Barrier-Penetrating Inhaled Nanoparticles for Enhanced Idiopathic Pulmonary Fibrosis Therapy. Adv. Funct. Mater..

[79] Shi T., Denney L., An H., Ho L.P., Zheng Y. (2021). Alveolar and lung interstitial macrophages: Definitions, functions, and roles in lung fibrosis. J. Leukoc. Biol..

[80] Jahnsen F.L., Gran E., Haye R., Brandtzaeg P. (2004). Human Nasal Mucosa Contains Antigen-Presenting Cells of Strikingly Different Functional Phenotypes. Am. J. Respir. Cell Mol. Biol..

[81] Patel B., Gupta N., Ahsan F. (2015). Particle engineering to enhance or lessen particle uptake by alveolar macrophages and to influence the therapeutic outcome. Eur. J. Pharm. Biopharm..

[82] Anselmo A.C., Zhang M.W., Kumar S., Vogus D.R., Menegatti S., Helgeson M.E., Mitragotri S. (2015). Elasticity of Nanoparticles Influences Their Blood Circulation, Phagocytosis, Endocytosis, and Targeting. ACS Nano.

[83] Mejías J.C., Roy K. (2019). In-vitro and in-vivo characterization of a multi-stage enzyme-responsive nanoparticle-in-microgel pulmonary drug delivery system. J. Control. Release.

[84] Teng Z., Yang J.K., Chen X.G., Liu Y. (2023). Intranasal Morphology Transformation Nanomedicines for Long-Term Intervention of Allergic Rhinitis. ACS Nano.

[85] Chen Y.R., Chen W., Xiang X.W., Deng L.F., Qian J.H., Cui W.G., Chen H. (2023). Pollen-Inspired Shell–Core Aerosol Particles Capable of Brownian Motion for Pulmonary Vascularization. Adv. Mater..

[86] Krishnamurthy S., Muthukumaran P., Jayakumar M.K.G., Lisse D., Masurkar N.D., Xu C., Chan J.M., Drum C.L. (2019). Surface protein engineering increases the circulation time of a cell membrane-based nanotherapeutic. Nanomedicine.

[87] Duan Y., Zhou J., Zhou Z., Zhang E., Yu Y., Krishnan N., Silva-Ayala D., Fang R.H., Griffiths A., Gao W. (2023). Extending the in vivo residence time of macrophage membrane-coated nanoparticles through genetic modification. Small.

[88] Thakkar H., Vaghela D., Patel B.P. (2021). Brain targeted intranasal in-situ gelling spray of paroxetine: Formulation, characterization and in-vivo evaluation. J. Drug Deliv. Sci. Technol..

[89] Bakshi S., Pandey P., Mohammed Y., Wang J., Sailor M.J., Popat A., Parekh H.S., Kumeria T. (2023). Porous silicon embedded in a thermoresponsive hydrogel for intranasal delivery of lipophilic drugs to treat rhinosinusitis. J. Control. Release.

[90] Cunha S., Swedrowska M., Bellahnid Y., Xu Z., Lobo J.M.S., Forbes B., Silva A.C. (2022). Thermosensitive in situ hydrogels of rivastigmine-loaded lipid-based nanosystems for nose-to-brain delivery: Characterisation, biocompatibility, and drug deposition studies. Int. J. Pharm..

[91] Knockenhauer K.E., Copeland R.A. (2023). The importance of binding kinetics and drug–target residence time in pharmacology. Br. J. Pharmacol..

[92] Wang H.R., Luo S., Xie M.X., Chen Z., Zhang Y.M., Xie Z.Q., Zhang Y.S., Zhang Y., Yang L., Wu F.H. (2024). ACE2 Receptor-Targeted Inhaled Nanoemulsions Inhibit SARS-CoV-2 and Attenuate Inflammatory Responses. Adv. Mater..

[93] Ryan G.M., Kaminskas L.M., Kelly B.D., Owen D.J., McIntosh M.P., Porter C.J. (2013). Pulmonary administration of PEGylated polylysine dendrimers: Absorption from the lung versus retention within the lung is highly size-dependent. Mol. Pharm..

[94] Anusha V., Umashankar M.S., Kumar Y.G. (2023). Pulsatile drug delivery system—An innovative method to treat chronotherapeutic diseases by synchronizing drug delivery with circadian rhythm. J. Appl. Pharm. Sci..

[95] Mura S., Nicolas J., Couvreur P. (2013). Couvreur, Stimuli-responsive nanocarriers for drug delivery. Nat. Mater..

[96] Wang X.H., Wang X.Y., Jin S.X., Muhammad N., Guo Z.J. (2019). Stimuli-Responsive Therapeutic Metallodrugs. Chem. Rev..

[97] Ferreira A.J., Cemlyn-Jones J., Cordeiro C.R. (2013). Nanoparticles, nanotechnology and pulmonary nanotoxicology. Rev. Port. Pneumol..

[98] Stewart P.S., Franklin M.J. (2008). Physiological heterogeneity in biofilms. Nat. Rev. Microbiol..

[99] Robba C., Siwicka-Gieroba D., Sikter A., Battaglini D., Dąbrowski W., Schultz M.J., De Jonge E., Grim C., Rocco P.R., Pelosi P. (2020). Pathophysiology and clinical consequences of arterial blood gases and pH after cardiac arrest. Intensive Care Med. Exp..

[100] Boedtkjer E., Pedersen S.F. (2020). The Acidic Tumor Microenvironment as a Driver of Cancer. Annu. Rev. Physiol..

[101] Hu Y., Ying J.Y. (2023). Reconfigurable A-motif, i-motif and triplex nucleic acids for smart pH-responsive DNA hydrogels. Mater. Today.

[102] Chen M.M., Hu J.X., Wang L.J., Li Y.R., Zhu C.H., Chen C., Shi M., Ju Z.C., Cao X.C., Zhang Z.Q. (2020). Targeted and redox-responsive drug delivery systems based on carbonic anhydrase IX-decorated mesoporous silica nanoparticles for cancer therapy. Sci. Rep..

[103] Karimi M., Ignasiak M.T., Chan B., Croft A.K., Radom L., Schiesser C.H., Pattison D.I., Davies M.J. (2016). Reactivity of disulfide bonds is markedly affected by structure and environment: Implications for protein modification and stability. Sci. Rep..

[104] Wang P.F., Gong Q.J., Hu J.B., Li X., Zhang X.J. (2021). Reactive Oxygen Species (ROS)-Responsive Prodrugs, Probes, and Theranostic Prodrugs: Applications in the ROS-Related Diseases. J. Med. Chem..

[105] Chung T.D., Linville R.M., Guo Z., Ye R., Jha R., Grifno G.N., Searson P.C. (2022). Effects of acute and chronic oxidative stress on the blood-brain barrier in 2D and 3D in vitro models. Fluids Barriers CNS.

[106] Liu Y.Q., Mao Y., Xu E., Jia H., Zhang S., Dawson V.L., Dawson T.M., Li Y.M., Zheng Z., He W. (2021). Nanozyme scavenging ROS for prevention of pathologic α-synuclein transmission in Parkinson’s disease. Nano Today.

[107] Xu S.T., Yang P., Qian K., Li Y.X., Guo Q., Wang P.Z., Meng R., Wu J., Cao J.X., Cheng Y.L. (2022). Modulating autophagic flux via ROS-responsive targeted micelles to restore neuronal proteostasis in Alzheimer’s disease. Bioact. Mater..

[108] Liu L., Liu M., Xiu J.Y., Zhang B.W., Hu H.Y., Qiao M.X., Chen D.W., Zhang J.L., Zhao X.L. (2023). Stimuli-responsive nanoparticles delivered by a nasal-brain pathway alleviate depression-like behavior through extensively scavenging ROS. Acta Biomater..

[109] Andresen T.L., Thompson D.H., Kaasgaard T. (2010). Enzyme-triggered nanomedicine: Drug release strategies in cancer therapy (Invited Review). Mol. Membr. Biol..

[110] Zhang R., Jing W.Q., Chen C., Zhang S.C., Abdalla M., Sun P., Wang G.Y., You W.J., Yang Z.M., Zhang J. (2022). Inhaled mRNA Nanoformulation with Biogenic Ribosomal Protein Reverses Established Pulmonary Fibrosis in a Bleomycin-Induced Murine Model. Adv. Mater..

[111] Dadmehr M., Mortezaei M., Korouzhdehi B. (2023). Dual mode fluorometric and colorimetric detection of matrix metalloproteinase MMP-9 as a cancer biomarker based on AuNPs@gelatin/AuNCs nanocomposite. Biosens. Bioelectron..

[112] Gou S.Q., Wang G.Y., Zou Y.N., Geng W.B., He T.T., Qin Z.Z., Che L.B., Feng Q., Cai K.Y. (2023). Non-Pore Dependent and MMP-9 Responsive Gelatin/Silk Fibroin Composite Microparticles as Universal Delivery Platform for Inhaled Treatment of Lung Cancer. Adv. Mater..

[113] Liu C., Xi L., Liu Y.H., Mak J.C.W., Mao S.R., Wang Z.P., Zheng Y. (2023). An Inhalable Hybrid Biomimetic Nanoplatform for Sequential Drug Release and Remodeling Lung Immune Homeostasis in Acute Lung Injury Treatment. ACS Nano.

[114] Driscoll K.E., Costa D.L., Hatch G., Henderson R., Oberdorster G., Salem H., Schlesinger R.B. (2000). Intratracheal Instillation as an Exposure Technique for the Evaluation of Respiratory Tract Toxicity: Uses and Limitations. Toxicol. Sci..

[115] Gungor S., Okyar A., Erturk-Toker S., Baktir G., Ozsoy Y. (2010). Ondansetron-loaded biodegradable microspheres as a nasal sustained delivery system: In vitro/in vivo studies. Pharm. Dev. Technol..

[116] Shringirishi M., Prajapati S.K., Mahor A., Alok S., Yadav P., Verma A. (2014). Nanosponges: A potential nanocarrier for novel drug delivery—A review. Asian Pac. J. Trop. Dis..

[117] Salem Y.Y., Hoti G., Sammour R.M.F., Caldera F., Cecone C., Matencio A., Shahiwala A.F., Trotta F. (2023). Preparation and evaluation of βcyclodextrin-based nanosponges loaded with Budesonide for pulmonary delivery. Int. J. Pharm..

[118] Nikolaou M., Avraam K., Kolokithas-Ntoukas A., Bakandritsos A., Lizal F., Misik O., Maly M., Jedelsky J., Savva I., Balanean F. (2021). Superparamagnetic electrospun microrods for magnetically-guided pulmonary drug delivery with magnetic heating. Mater. Sci. Eng. C.

[119] Wang M., Hou J., Yu D.G., Li S., Zhu J., Chen Z. (2020). Electrospun tri-layer nanodepots for sustained release of acyclovir. J. Alloys Compd..

[120] Wang Y., Yu D.G., Liu Y., Liu Y.N. (2022). Progress of Electrospun Nanofibrous Carriers for Modifications to Drug Release Profiles. J. Funct. Biomater..

[121] Narayanan S., Pavithran M., Viswanath A., Narayanan D., Mohan C.C., Manzoor K., Menon D. (2014). Sequentially releasing dual-drug-loaded PLGA–casein core/shell nanomedicine: Design, synthesis, biocompatibility and pharmacokinetics. Acta Biomater..

[122] Velusamy A., Sharma R., Rashid S.A., Ogasawara H., Salaita K. (2024). DNA mechanocapsules for programmable piconewton responsive drug delivery. Nat. Commun..

[123] Oh J.Y., Kim H.S., Palanikumar L., Go E.M., Jana B., Park S.A., Kim H.Y., Kim K., Seo J.K., Kwak S.K. (2018). Cloaking nanoparticles with protein corona shield for targeted drug delivery. Nat. Commun..

[124] Srinivasarao M., Low P.S. (2017). Ligand-Targeted Drug Delivery. Chem. Rev..

[125] Liu M.R., Lutz H., Zhu D., Huang K., Li Z.H., Dinh P.U.C., Gao J.Q., Zhang Y., Cheng K. (2021). Bispecific Antibody Inhalation Therapy for Redirecting Stem Cells from the Lungs to Repair Heart Injury. Adv. Sci..

[126] Tseng C.L., Wu S.Y.H., Wang W.H., Peng C.L., Lin F.H., Lin C.C., Young T.H., Shieh M.J. (2008). Targeting efficiency and biodistribution of biotinylated-EGF-conjugated gelatin nanoparticles administered via aerosol delivery in nude mice with lung cancer. Biomaterials.

[127] Rosière R., Van Woensel M., Gelbcke M., Mathieu V., Hecq J., Mathivet T., Vermeersch M., Van Antwerpen P., Amighi K., Wauthoz N. (2018). New Folate-Grafted Chitosan Derivative To Improve Delivery of Paclitaxel-Loaded Solid Lipid Nanoparticles for Lung Tumor Therapy by Inhalation. Mol. Pharm..

[128] Huang X., Chisholm J., Zhuang J., Xiao Y., Duncan G., Chen X., Suk J.S., Hanes J. (2017). Protein nanocages that penetrate airway mucus and tumor tissue. Proc. Natl. Acad. Sci. USA.

[129] Yu Y.L., Ni M.J., Zheng Y.X., Huang Y. (2024). Airway epithelial-targeted nanoparticle reverses asthma in inhalation therapy. J. Control. Release.

[130] Kim M.K., Lee Y.K., Lee M.H. (2021). Hypoxia-specific anti-RAGE exosomes for nose-to-brain delivery of anti-miR-181a oligonucleotide in an ischemic stroke model. Nanoscale.

[131] Peng H., Li Y., Ji W.H., Zhao R.C., Lu Z.G., Shen J., Wu Y.Y., Wang J.Z., Hao Q.L., Wang J.W. (2022). Intranasal Administration of Self-Oriented Nanocarriers Based on Therapeutic Exosomes for Synergistic Treatment of Parkinson’s Disease. ACS Nano.

[132] Li J.X., Peng H., Zhang W., Li M.Z., Wang N., Peng C., Zhang X.Y., Li Y. (2023). Enhanced Nose-to-Brain Delivery of Combined Small Interfering RNAs Using Lesion-Recognizing Nanoparticles for the Synergistic Therapy of Alzheimer’s Disease. ACS Appl. Mater. Interfaces.

[133] Tang L., Zhang R., Wang Y.S., Zhang X.Y., Yang Y.L., Zhao B.Y., Yang L. (2023). A simple self-assembly nanomicelle based on brain tumor-targeting peptide-mediated siRNA delivery for glioma immunotherapy via intranasal administration. Acta Biomater..

[134] Thanuja M.Y., Anupama C., Ranganath S.H. (2018). Bioengineered cellular and cell membrane-derived vehicles for actively targeted drug delivery: So near and yet so far. Adv. Drug Deliv. Rev..

[135] Wang Q., Li T., Yang J., Zhao Z.N., Tan K.Y., Tang S.W., Wan M.M., Mao C. (2022). Engineered Exosomes with Independent Module/Cascading Function for Therapy of Parkinson’s Disease by Multistep Targeting and Multistage Intervention Method. Adv. Mater..

[136] Pan J.M., Wang Z.H., Huang X.H., Xue J., Zhang S.L., Guo X., Zhou S.B. (2023). Bacteria-Derived Outer-Membrane Vesicles Hitchhike Neutrophils to Enhance Ischemic Stroke Therapy. Adv. Mater..

[137] Wang S.Y., Yang L.F., He W.Y., Zheng M., Zou Y. (2024). Cell Membrane Camouflaged Biomimetic Nanoparticles as a Versatile Platform for Brain Diseases Treatment. Small Methods.

[138] Zhang K., Long Y.Y., Li S.T., Zhao Y.L., Han H.Y. (2024). Inhalable biomimetic nanomotor for pulmonary thrombus therapy. Nano Today.

[139] Popowski K.D., Moatti A., Scull G., Silkstone D., Lutz H., Abad B.L.J., George A., Belcher E., Zhu D., Mei X. (2022). Inhalable dry powder mRNA vaccines based on extracellular vesicles. Matter.

[140] Zheng B., Peng W.C., Guo M.M., Huang M.Q., Gu Y.X., Wang T., Ni G.J., Ming D. (2021). Inhalable nanovaccine with biomimetic coronavirus structure to trigger mucosal immunity of respiratory tract against COVID-19. Chem. Eng. J..

[141] D’Addio S.M., Prud’Homme R.K. (2011). Controlling drug nanoparticle formation by rapid precipitation. Adv. Drug Deliv. Rev..

[142] Abdelwahed W., Degobert G., Stainmesse S., Fessi H. (2006). Freeze-drying of nanoparticles: Formulation, process and storage considerations. Adv. Drug Deliv. Rev..

[143] Komalla V., Wong C.Y.J., Sibum I., Muellinger B., Nijdam W., Chaugule V., Soria J., Ong H.X., Buchmann N.A., Traini D. (2023). Advances in soft mist inhalers. Expert Opin. Drug Deliv..

[144] Wang X., Gadhave D., Chauhan G., Gupta V. (2024). Development and characterization of inhaled nintedanib-loaded PLGA nanoparticles using scalable high-pressure homogenization technique. J. Drug Deliv. Sci. Technol..

[145] Solé-Porta A., Areny-Balagueró A., Camprubí-Rimblas M., Fernández E.F., O’Sullivan A., Giannoccari R., MacLoughlin R., Closa D., Artigas A., Roig A. (2024). Efficient Nebulization and Pulmonary Biodistribution of Polymeric Nanocarriers in an Acute Lung Injury Preclinical Model. Small Sci..

[146] Paunovska K., Gil C.J., Lokugamage M.P., Sago C.D., Sato M., Lando G.N., Castro M.G., Bryksin A.V., Dahlman J.E. (2018). Analyzing 2000 in vivo drug delivery data points reveals cholesterol structure impacts nanoparticle delivery. ACS Nano.

[147] Ball R.L., Hajj K.A., Vizelman J., Bajaj P., Whitehead K.A. (2018). Lipid nanoparticle formulations for enhanced co-delivery of siRNA and mRNA. Nano Lett..

[148] Lokugamage M.P., Vanover D., Beyersdorf J., Hatit M.Z.C., Rotolo L., Echeverri E.S., Peck H.E., Ni H., Yoon J.-K., Kim Y. (2021). Optimization of lipid nanoparticles for the delivery of nebulized therapeutic mRNA to the lungs. Nat. Biomed. Eng..

[149] Szabová J., Mišík O., Havlíková M., Lízal F., Mravec F. (2021). Influence of liposomes composition on their stability during the nebulization process by vibrating mesh nebulizer. Colloids Surf. B Biointerfaces.

[150] Jiang A.Y., Witten J., Raji I.O., Eweje F., Macisaac C., Meng S., Oladimeji F.A., Hu Y., Manan R.S., Langer R. (2024). Combinatorial development of nebulized mRNA delivery formulations for the lungs. Nat. Nanotechnol..

[151] Zhang H., Leal J., Soto M.R., Smyth H.D., Ghosh D. (2020). Aerosolizable lipid nanoparticles for pulmonary delivery of mRNA through design of experiments. Pharmaceutics.

[152] Malamatari M., Charisi A., Malamataris S., Kachrimanis K., Nikolakakis I. (2020). Spray Drying for the Preparation of Nanoparticle-Based Drug Formulations as Dry Powders for Inhalation. Processes.

[153] Chang Z.Y., Wang W.H., Huang Z.W., Huang Y., Wu C.B., Pan X. (2023). Lecithin Reverse Micelle System is Promising in Constructing Carrier Particles for Protein Drugs Encapsulated Pressurized Metered-Dose Inhalers. Adv. Ther..

[154] Lockhart J.N., Beezer D.B., Stevens D.M., Spears B.R., Harth E. (2016). One-pot polyglycidol nanogels via liposome master templates for dual drug delivery. J. Control. Release.

[155] Nele V., Schutt C.E., Wojciechowski J.P., Kit-Anan W., Doutch J.J., Armstrong J.P.K., Stevens M.M. (2020). Ultrasound-Triggered Enzymatic Gelation. Adv. Mater..

[156] Liao S.C., Ting C.W., Chiang W.H. (2020). Functionalized polymeric nanogels with pH-sensitive benzoic-imine cross-linkages designed as vehicles for indocyanine green delivery. J. Colloid Interface Sci..

[157] Szebeni J., Kiss B., Bozó T., Turjeman K., Levi-Kalisman Y., Barenholz Y., Kellermayer M. (2023). Insights into the Structure of Comirnaty Covid-19 Vaccine: A Theory on Soft, Partially Bilayer-Covered Nanoparticles with Hydrogen Bond-Stabilized mRNA–Lipid Complexes. ACS Nano.

[158] Petralito S., Paolicelli P., Nardoni M., Trilli J., Di Muzio L., Cesa S., Relucenti M., Matassa R., Vitalone A., Adrover A. (2020). Gelation of the internal core of liposomes as a strategy for stabilization and modified drug delivery I. Physico-chemistry study. Int. J. Pharm..

[159] Yuan T.Y., Shao Y., Zhou X., Liu Q., Zhu Z.C., Zhou B.N., Dong Y.C., Stephanopoulos N., Gui S., Yan H. (2021). Highly permeable DNA supramolecular hydrogel promotes neurogenesis and functional recovery after completely transected spinal cord injury. Adv. Mater..

[160] Wei X.N., Li Y.J., Cheng X.D., Wen Y.X., Yuan W., Chen R.F., Meng S.W., Lu X.G., Yu Z.Y., Xu L.J. (2023). Increase Nebulization Stability of Lipid Nanoparticles by Integrating a DNA Supramolecular Hydrogel. ACS Macro Lett..

[161] Manconi M., Manca M.L., Valenti D., Escribano E., Hillaireau H., Fadda A.M., Fattal E. (2017). Chitosan and hyaluronan coated liposomes for pulmonary administration of curcumin. Int. J. Pharm..

[162] Wang H.Z., Yuan Y., Qin L., Yue M.M., Xue J.W., Cui Z.X., Zhan X.G., Gai J.Y., Zhang X., Guan J. (2024). Tunable rigidity of PLGA shell-lipid core nanoparticles for enhanced pulmonary siRNA delivery in 2D and 3D lung cancer cell models. J. Control. Release.

[163] Dhayanandamoorthy Y., Antoniraj M.G., Kandregula C.A.B., Kandasamy R. (2020). Aerosolized hyaluronic acid decorated, ferulic acid loaded chitosan nanoparticle: A promising asthma control strategy. Int. J. Pharm..

[164] Thakur A., Ingvarsson P.T., Schmidt S.T., Rose F., Andersen P., Christensen D., Foged C. (2018). Immunological and physical evaluation of the multistage tuberculosis subunit vaccine candidate H56/CAF01 formulated as a spray-dried powder. Vaccine.

[165] Ye T., Jiao Z.G., Li X., He Z.L., Li Y.Y., Yang F.M., Zhao X., Wang Y.C., Huang W.J., Qin M. (2023). Inhaled SARS-CoV-2 vaccine for single-dose dry powder aerosol immunization. Nature.

[166] Van Holsbeke C., Marshall J., De Backer J., Vos W. (2014). Median mass aerodynamic diameter (MMAD) and fine particle fraction (FPF): Influence on lung deposition?. Eur. Respir. J..

[167] Hickey A.J., Stewart I.E. (2022). Inhaled antibodies: Quality and performance considerations. Hum. Vaccin. Immunother..

[168] Jones N. (2001). The nose and paranasal sinuses physiology and anatomy. Adv. Drug Deliv. Rev..

[169] The Food and Drug Administration (2003). Bioavailability and Bioequivalence Studies for Nasal Aerosols and Nasal Sprays for Local Action. https://www.fda.gov/regulatory-information/search-fda-guidance-documents/bioavailability-and-bioequivalence-studies-nasal-aerosols-and-nasal-sprays-local-action.

[170] Grmaš J., Stare K., Božič D., Injac R., Dreu R. (2017). Elucidation of Formulation and Delivery Device-Related Effects on In Vitro Performance of Nasal Spray with Implication to Rational Product Specification Identification. J. Aerosol Med. Pulm. Drug Deliv..

[171] Edwards D.A., Hanes J., Caponetti G., Hrkach J., Ben-Jebria A., Eskew M.L., Mintzes J., Deaver D., Lotan N., Langer R. (1997). Large porous particles for pulmonary drug delivery. Science.

[172] Zhu L.F., Li M., Liu X.Y., Du L.N., Jin Y.G. (2017). Inhalable oridonin-loaded poly(lactic-co-glycolic) acid large porous microparticles for in situ treatment of primary non-small cell lung cancer. Acta Pharm. Sin. B.

[173] Pacheco J.E.C., Yalovenko T., Riaz A., Kotov N., Davids C., Persson A., Falkman P., Feiler A., Godaly G., Johnson C.M. (2024). Inhalable porous particles as dual micro-nano carriers demonstrating efficient lung drug delivery for treatment of tuberculosis. J. Control. Release.

[174] Zhang L., Yang L., Zhang X., Jiaqi L., Fan L., Beck-Broichsitter M., Zhang X., Muenster U., Wang X., Zhao J. (2018). Sustained therapeutic efficacy of budesonide-loaded chitosan swellable microparticles after lung delivery: Influence of in vitro release, treatment interval and dose. J. Control. Release.

[175] Behrend-Keim B., Castro-Muñoz A., Monrreal-Ortega L., Ávalos-León B., Campos-Estrada C., Smyth H.D.C., Bahamondez-Canas T.F., Moraga-Espinoza D. (2023). The forgotten material: Highly dispersible and swellable gelatin-based microspheres for pulmonary drug delivery of cromolyn sodium and ipratropium bromide. Int. J. Pharm..

[176] Pablo E., Fernández-García R., Ballesteros M.P., Torrado J.J., Serrano D.R. (2017). Nebulised antibiotherapy: Conventional versus nanotechnology-based approaches, is targeting at a nano scale a difficult subject?. Ann. Transl. Med..

[177] Cao J., Xu Y., Zhang J., Fang T., Wu F., Zhen Y., Yu X., Liu Y., Li J., Wang D. (2024). “Nano-in-Micro” Structured Dry Powder Inhalers for pulmonary delivery: Advances and challenges. J. Drug Deliv. Sci. Technol..

[178] Wang Q.Y., Shen Y., Mi G.J., He D.S., Zhang Y., Xiong Y.R., Webster T.J., Tu J.S. (2020). Fumaryl diketopiperazine based effervescent microparticles to escape macrophage phagocytosis for enhanced treatment of pneumonia via pulmonary delivery. Biomaterials.

[179] Zhang X., Zhao Z., Cui Y., Liu F., Huang Z., Huang Y., Zhang R., Freeman T., Lu X., Pan X. (2019). Effect of powder properties on the aerosolization performance of nanoporous mannitol particles as dry powder inhalation carriers. Powder Technol..

[180] Abdelaziz H.M., Elzoghby A.O., Helmy M.W., Abdelfattah E.-Z.A., Fang J.-Y., Samaha M.W., Freag M.S. (2020). Inhalable Lactoferrin/Chondroitin-Functionalized Monoolein Nanocomposites for Localized Lung Cancer Targeting. ACS Biomater. Sci. Eng..

[181] Fernández-Paz E., Fernández-Paz C., Barrios-Esteban S., Santalices I., Csaba N., Remuñán-López C. (2022). Dry powders containing chitosan-based nanocapsules for pulmonary administration: Adjustment of spray-drying process and in vitro evaluation in A549 cells. Powder Technol..

[182] Zhang X.J., Zhou Y., Wang G.L., Zhao Z.Y., Jiang Z.X., Cui Y.T., Yue X., Huang Z.W., Huang Y., Pan X. (2022). Co-spray-dried poly-L-lysine with L-leucine as dry powder inhalations for the treatment of pulmonary infection: Moisture-resistance and desirable aerosolization performance. Int. J. Pharm..

[183] Ali M.E., Lamprecht A. (2017). Spray freeze drying as an alternative technique for lyophilization of polymeric and lipid-based nanoparticles. Int. J. Pharm..

[184] Fukushige K., Tagami T., Naito M., Goto E., Hirai S., Hatayama N., Yokota H., Yasui T., Baba Y., Ozeki T. (2020). Developing spray-freeze-dried particles containing a hyaluronic acid-coated liposome–protamine–DNA complex for pulmonary inhalation. Int. J. Pharm..

[185] Rospond B., Krakowska A., Muszyńska B., Opoka W. (2022). The history, current state and perspectives of aerosol therapy. Acta Pharm..

[186] Häußermann S., Arendsen L.J., Pritchard J.N. (2022). Smart dry powder inhalers and intelligent adherence management. Adv. Drug Deliv. Rev..

[187] Shirley M. (2019). Amikacin Liposome Inhalation Suspension: A Review in Mycobacterium avium Complex Lung Disease. Drugs.

[188] Kim J., Jozić A., Bloom E., Jones B., Marra M., Murthy N.T.V., Eygeris Y., Sahay G. (2024). Microfluidic Platform Enables Shearless Aerosolization of Lipid Nanoparticles for mRNA Inhalation. ACS Nano.

[189] Cees J.M., Kilian V.E., Rob J.D., Albert P., Timo B., Selina L., Wim A.E., van Wijnbergen K., van Hamme J.L., Daniel B. (2023). Low energy nebulization preserves integrity of SARS-CoV-2 mRNA vaccines for respiratory delivery. Sci. Rep..

[190] Yeo L.Y., Friend J.R., McIntosh M.P., Meeusen E.N., Morton D.A. (2010). Ultrasonic nebulization platforms for pulmonary drug delivery. Expert Opin. Drug Deliv..

[191] Cortez-Jugo C., Masoumi S., Chan P.P.Y., Friend J., Yeo L. (2022). Nebulization of siRNA for inhalation therapy based on a microfluidic surface acoustic wave platform. Ultrason. Sonochem..

[192] Klein D.M., Poortinga A., Verhoeven F.M., Bonn D., Bonnet S., van Rijn C.J. (2021). Degradation of lipid based drug delivery formulations during nebulization. Chem. Phys..

[193] Farnoud A., Baumann I., Rashidi M.M., Schmid O., Gutheil E. (2020). Simulation of patient-specific bi-directional pulsating nasal aerosol dispersion and deposition with clockwise 45°and 90°nosepieces. Comput. Biol. Med..

[194] Le Guellec S., Ehrmann S., Vecellio L. (2021). In vitro–in vivo correlation of intranasal drug deposition. Adv. Drug Deliv. Rev..

[195] Pina Costa C., Nodilo L.N., Silva R., Martins E., Zadravec D., Kalogjera L., Moreira J.N., Lobo J.M.S., Hafner A., Silva A.C. (2023). In situ hydrogel containing diazepam-loaded nanostructured lipid carriers (DZP-NLC) for nose-to-brain delivery: Development, characterization and deposition studies in a 3D-printed human nasal cavity model. Int. J. Pharm..

[196] Maaz A., Blagbrough I.S., De Bank P.A. (2024). A Cell-Based Nasal Model for Screening the Deposition, Biocompatibility, and Transport of Aerosolized PLGA Nanoparticles. Mol. Pharm..

[197] Djupesland P.G., Skretting A. (2012). Nasal Deposition and Clearance in Man: Comparison of a Bidirectional Powder Device and a Traditional Liquid Spray Pump. J. Aerosol Med. Pulm. Drug Deliv..

[198] Vu H.D., Vu T.H., Mai N.L., Pande D.C., Dao D.V., Rehm B.H., Nguyen N.T., Grant G.D., Tran C.D., Zhu Y. (2024). In-flight electro-neutralisation electrospray for pulmonary drug delivery. Nano Today.

[199] Chaugule V., Reis L.G.D., Fletcher D.F., Young P.M., Traini D., Soria J. (2024). A counter-swirl design concept for dry powder inhalers. Int. J. Pharm..

[200] Lee H.J., Kwon I.H., Lee H.G., Kwon Y.B., Woo H.M., Cho S.M., Choi Y.W., Chon J., Kim K., Kim D.W. (2018). Spiral mouthpiece design in a dry powder inhaler to improve aerosolization. Int. J. Pharm..

